# Identification of the Virulence Landscape Essential for *Entamoeba histolytica* Invasion of the Human Colon

**DOI:** 10.1371/journal.ppat.1003824

**Published:** 2013-12-19

**Authors:** Roman Thibeaux, Christian Weber, Chung-Chau Hon, Marie-Agnès Dillies, Patrick Avé, Jean-Yves Coppée, Elisabeth Labruyère, Nancy Guillén

**Affiliations:** 1 Institut Pasteur, Unité Biologie Cellulaire du Parasitisme, Paris, France; 2 INSERM U786, Paris, France; 3 Université de Versailles Saint-Quentin-en-Yvelines, Versailles, France; 4 Institut Pasteur, Transcriptome et Epigénome, Département Génomes et Génétique, Paris, France; 5 Institut Pasteur, Unité Histopathologie Humaine et Modèles Animaux, Paris, France; University of Virginia Health System, United States of America

## Abstract

*Entamoeba histolytica* is the pathogenic amoeba responsible for amoebiasis, an infectious disease targeting human tissues. Amoebiasis arises when virulent trophozoites start to destroy the muco-epithelial barrier by first crossing the mucus, then killing host cells, triggering inflammation and subsequently causing dysentery. The main goal of this study was to analyse pathophysiology and gene expression changes related to virulent (i.e. HM1:IMSS) and non-virulent (i.e. Rahman) strains when they are in contact with the human colon. Transcriptome comparisons between the two strains, both in culture conditions and upon contact with human colon explants, provide a global view of gene expression changes that might contribute to the observed phenotypic differences. The most remarkable feature of the virulent phenotype resides in the up-regulation of genes implicated in carbohydrate metabolism and processing of glycosylated residues. Consequently, inhibition of gene expression by RNA interference of a glycoside hydrolase (β-amylase absent from humans) abolishes mucus depletion and tissue invasion by HM1:IMSS. In summary, our data suggest a potential role of carbohydrate metabolism in colon invasion by virulent *E. histolytica*.

## Author Summary

*Entamoeba histolytica* is an intestinal parasite which displays diverse phenotypes with respect to pathogenesis in the human colon. Trophozoites can remain as commensal, without causing evident intestinal damage, or they can destroy the colonic mucosa leading to amoebiasis. Using human colon explants and transcriptome analysis, we investigated the gene expression profile of two *E. histolytica* strains (virulent and non-virulent) during their contact with the intestinal mucus to gain insights into the molecular basis responsible for amoebic divergent phenotypes. Our results suggest that the virulent *E. histolytica*, when in contact with the intestinal barrier, specifically increases the rate of gene transcription for enzymes necessary to exploits the carbohydrate resources present in the human colon. Using RNA interference methodologies to knockdown gene expression, our data revealed the potential role of amoebic β-amylase (a glycosydase) in colon invasion and mucus depletion. Our data implies that the ability of an *E. histolytica* strain to exploit the carbohydrate resources might affect its ability to invasion the intestine.

## Introduction

In the human colon, mucus acts as a lubricant facilitating the passage of digestive content, protects the underlying epithelium from mechanical stress, and provides a protective barrier against pathogens. Mucin 2 (MUC2) is the major component of the mucus layer. MUC2 is a heavily glycosylated protein, containing more than 100 different glycan chain variants which are responsible for approximately 80% of the MUC2 mass [1]. The extensive glycosylation of MUC2 provides protection to resist proteolytic activities. The MUC2-related glycans also represent a potential carbon source for microbiota nutrition, mainly in the distal colon where the availability of free carbohydrates is limited. For instance, intestinal commensal bacteria express genes involved in the biodegradation of complex sugars and glycans present in dietary fibers [2] or genes important for degrading the endogenous pool of host glycans, the last offering a permanent nutrient source for the gut microbiota [3]. During infection, pathogens and resident microbiota compete for nutritional metabolites present in the intestinal lumen and therefore changes in carbon availability may alter the equilibrium in the colon ecosystem contributing to the susceptibility to infection.

*Entamoeba histolytica* is a protozoan parasite residing in the human colon where it feeds on bacteria. In some cases, trophozoites invade the tissue leading to intestinal amoebiasis and, in rare cases, to hepatic amoebiasis. *E. histolytica* infection is a persistent and worldwide disease that is the third leading cause of mortality due to a protozoan [4]. Most infections with this parasite are asymptomatic since only ∼20% of the cases develop intestinal amoebiasis, which are characterized by colonic mucosa invasion and tissue destruction. Trophozoites have been isolated from symptomatic and asymptomatic patients. *E. histolytica* HM1:IMSS, isolated from a patient suffering from amoebic dysentery, is a virulent strain routinely used to reproduce the main features of intestinal [5] and hepatic amoebiasis [6] in experimental models. Another strain, *E. histolytica* Rahman, was isolated from an asymptomatic carrier, it is unable to growth in animals due to its inherent phenotype and is classically referred as a non-virulent strain [7]. Analysis of the 5.8S rRNA sequences indicates that Rahman belongs to *E. histolytica* species [8], nonetheless, the Rahman strain presents a reduced cytotoxicity towards epithelial cells *in vitro* [9], does not form liver abscesses in animal models [10], exhibits defects in phagocytosis, and shows significantly reduced virulence in a human intestinal xenograft model of amoebic colitis [11]. A genomic hybridization study comparing HM1:IMSS and Rahman strains revealed that only 5 out of 1817 genes studied are significantly divergent [12]. At the protein level, important differences in Rahman have nevertheless been described including a truncated glycan chain of the proteophosphoglycan coating the surface [13], a decreased level of both peroxiredoxin [11] and the light subunit of the Gal/GalNAc lectin [9]. Several studies have also attempted to identify genes whose expression correlates with a virulent phenotype by comparing the transcriptomes of both strains under culture conditions [14], [15]. Although they highlighted changes in multiple pathways during parasite axenic growth, no clear explanation has been given to account for their differences in virulence.

To gain insights into the molecular basis of phenotypic differences between *E. histolytica* HM1:IMSS and Rahman during their interaction with the intestine, we took advantage of the human colon *ex vivo* model of amoebiasis [5]. Their interaction with the human colon explants was investigated by analysing the morphological changes of the mucosa architecture. We then performed a gene expression analysis for each strain and made comparisons between their transcriptomes. We identified (i) genes that are constitutively expressed in each strain in the two different environments (i.e. in axenic culture or human colon explants), (ii) transcripts specifically upregulated in each strain upon contact with human colon explants, and (iii) transcripts commonly modulated in both strains upon contact with human colon explants.

Genes encoding glycolytic enzymes, carbohydrate catabolism enzymes, and genes characterized as virulent factors were identified and exclusively upregulated in HM1:IMSS upon contact with human colon explants. In particular, one of the most upregulated genes in HM1:IMSS is β-amylase, a glycoside hydrolase absent in humans. The potential role of β-amylase in colon invasion was further investigated by knocking down of its encoding gene using double-stranded RNA (dsRNA). Parasites treated with dsRNA were unable to deplete the mucus and subsequently invade the human colon explants. Altogether, our data provides a novel view of how *E. histolytica* crosses the intestinal barrier and suggests new avenues to understand amoebic pathogenicity.

## Results

### *Entamoeba histolytica* Rahman strain binds to, but does not deplete the human mucus layer

**Figure 1:**
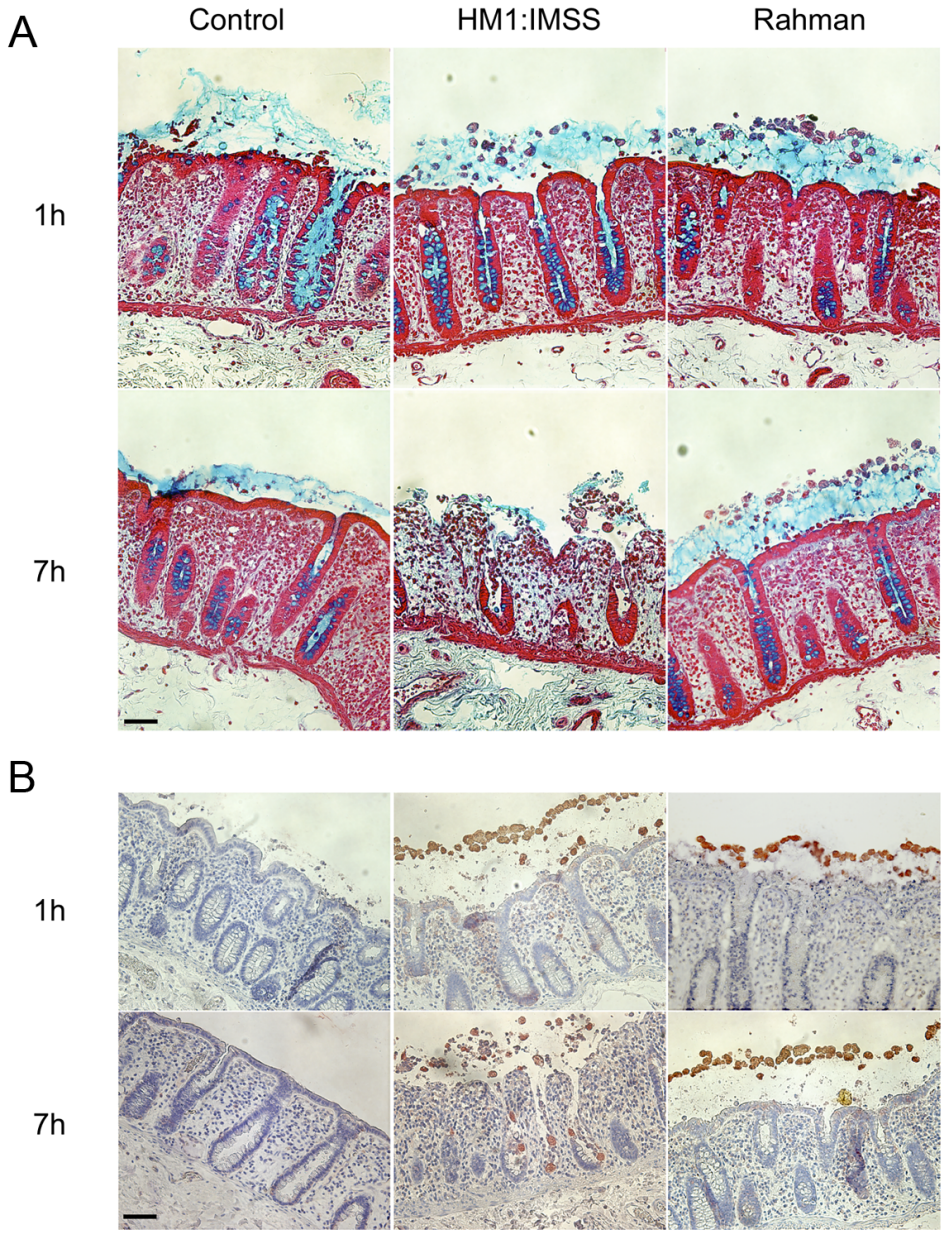
Human colon explants incubated with *E. histolytica* HM1:IMSS or Rahman trophozoites. Longitudinal tissue sections of colon explants incubated during 1 or 7*E. histolytica* HM1:IMSS or Rahman trophozoites. The upper panel (A) corresponds to the alcian blue staining of the mucus in the top followed by staining of the tissue where the epithelial cells and the crypts of Liberkün (counterstain in red with Safranin) are visible. The lower panel (B) corresponds to immunohistochemistry revealing the presence of trophozoites in the top by immunostaining for the Gal/GalNAc lectin and the tissue by counterstaining with Hematoxylin/Eosin (bleu). Note the presence of Rahman trophozoites on top of the mucus layer even after 7 h of incubation and the massive destruction of the mucosa in the presence of HM1:IMSS. Scale bar = 50 µm.

To investigate the phenotypic differences between the virulent HM1:IMSS and non-virulent Rahman strains during their interactions with the human colon explants, we monitored the *ex-vivo* invasion of human colon explants from six patients. After 1 or 7 hours (h) of incubation with trophozoites, colon fragments were fixed for histological analysis and longitudinal sections of the tissues were prepared and examined for mucus integrity (Figure 1A) and tissue invasion (Figure 1B). After 1 h of incubation, the protective mucus layer remains intact in all three conditions (Figure 1A). After 7 h of incubation, we observed tissue penetration by the HM1:IMSS trophozoites with a strong depletion of the mucus layer (Figure 1A). Trophozoites were then localized by immunostaining for the Gal/GalNAc lectin (Figure 1B). The HM1:IMSS trophozoites degraded the intestinal epithelium and penetrated into the mucosa as described previously [5], [16]. In contrast to these findings, after 7 h of incubation with the Rahman strain, trophozoites were still at the surface of the explant, no penetration of the mucosa was observed, and the tissue structure remains intact (Figure 1B). We utilized video microscopy to monitor Rahman trophozoites on the explants to ensure that they were still viable during the incubation (Video S1).

### Rationale for transcriptome comparisons between HM1:IMSS and Rahman strains in axenic culture and upon contact with human colon explants

**Figure 2:**
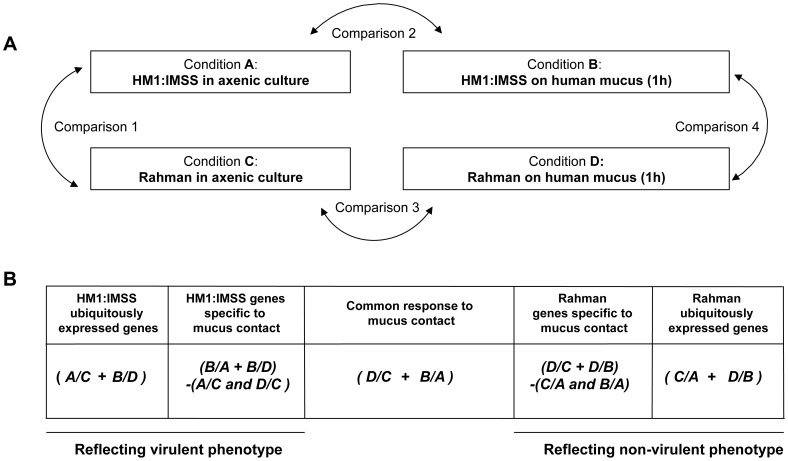
Experimental design and strategy for microarray data analysis. A. Scheme representing the four experimental conditions and transcriptome comparisons. B. Cross-comparative analysis accomplished to identify strain-specific and common responses under the two conditions tested. The transcriptome data were processed as indicated in order to identify gene expression profiles associated with the strain and the colon explant contact. A/B designates the pool of genes overexpressed in condition A compared to condition B. + means that the common overexpressed genes of the comparisons are kept, whereas - indicates genes that were discarded. Note that the profiles obtained by this strategy are composed of genes with increase transcript levels in a particular strain versus the other.

To identify gene expression specifically modulated in HM1:IMSS and Rahman strains upon contact with the human colon explants, total RNA was purified from trophozoites in axenic culture and after contact for 1 h with the human colon explants, a time where virulent trophozoites begin to penetrate through the mucus layer of the colonic tissue [5]. The experiment was conducted on six independent human colon explants from six patients and therefore six biological replicates. Each explant was cut into three pieces, the first was incubated with HM1:IMSS trophozoites, the second was incubated with Rahman trophozoites and the third was incubated without trophozoites (as a control for pathophysiology). RNA was purified from the amoebic samples in contact with the tissue as well as from amoebic samples growing in *in vitro* culture. As a control, RNA was also purified from trophozoites of both strains incubated in Krebs buffer only (the medium for incubation with the human colon explants). RNA was then reverse-transcribed and hybridized with whole genome cDNA microarrays (EH-IP2008, Agilent technologies) as described previously [17]. A total of 54 hybridizations were performed. For transcriptome data analysis we adopted a step-by-step strategy. First, we performed pairwise comparisons (Figure 2A) with statistics computed for each gene and each condition to identify the transcriptome differences between HM1:IMSS and Rahman strains under axenic culture (Comparison 1) and upon contact with the human colon explants (Comparison 4). The pairwise comparisons also identified transcriptome responses specific for each strain when comparing the human colon explant to the respective profiles in axenic culture (Comparisons 2 and 3). The genes that were commonly modulated in TYI or Krebs buffer only were eliminated from the analysis (Table S1 and S2).

In the second part of the analysis, we used a nested statistical approach (Limma package [18]) where values were tested across the comparisons as indicated in Figure 2B. The gene expression profile of ubiquitously expressed genes for HM1:IMSS was defined by the genes upregulated during both in axenic culture and upon contact with the human colon explants, compared to Rahman (Conditions (A/C+B/D)). Notice that in this analysis genes downregulated in HM1:IMSS were also considered since these genes became upregulated in Rahman. Similarly, the gene expression profile of ubiquitously expressed genes for Rahman was obtained by detecting genes upregulated in axenic culture and upon contact with the human colon explants compared to HM1:IMSS (Conditions (C/A+D/B)). Since both strains bind to the mucus, we searched for a gene expression profile reflecting their common responses the mucus contact by comparing the upregulated genes shared by both strains upon contact with the human colon explants compared to axenic culture (Figure 2B, Conditions (D/C+B/A)). Furthermore, we established the gene expression profile of HM1:IMSS genes specifically expressed during mucus contact, composed of upregulated genes in HM1:IMSS upon contact with the human colon explants compared (i) to the axenic culture and (ii) to Rahman upon contact with the human colon explants. We removed genes upregulated in HM1:IMSS in axenic culture compared to both Rahman in axenic culture and Rahman upon contact with the human colon explants (Conditions (B/A+B/D)−(A/C and D/C)). Analogously, we obtained the gene expression profile of Rahman genes specifically expressed during mucus contact, composed of upregulated genes in the Rahman upon contact with the human colon explants as compared (i) to the axenic culture and (ii) to HM1:IMSS upon contact with the human colon explants. We removed genes that were upregulated in HM1:IMSS in the axenic culture and upon contact with the human colon explants (Conditions (D/C+D/B)−(C/A and B/A)). Overall the combined analysis established the gene expression profiles characteristic of the virulent and non-virulent phenotypes.

### Identification of transcriptome profiles of *E. histolytica* HM1:IMSS and Rahman strains

**Figure 3:**
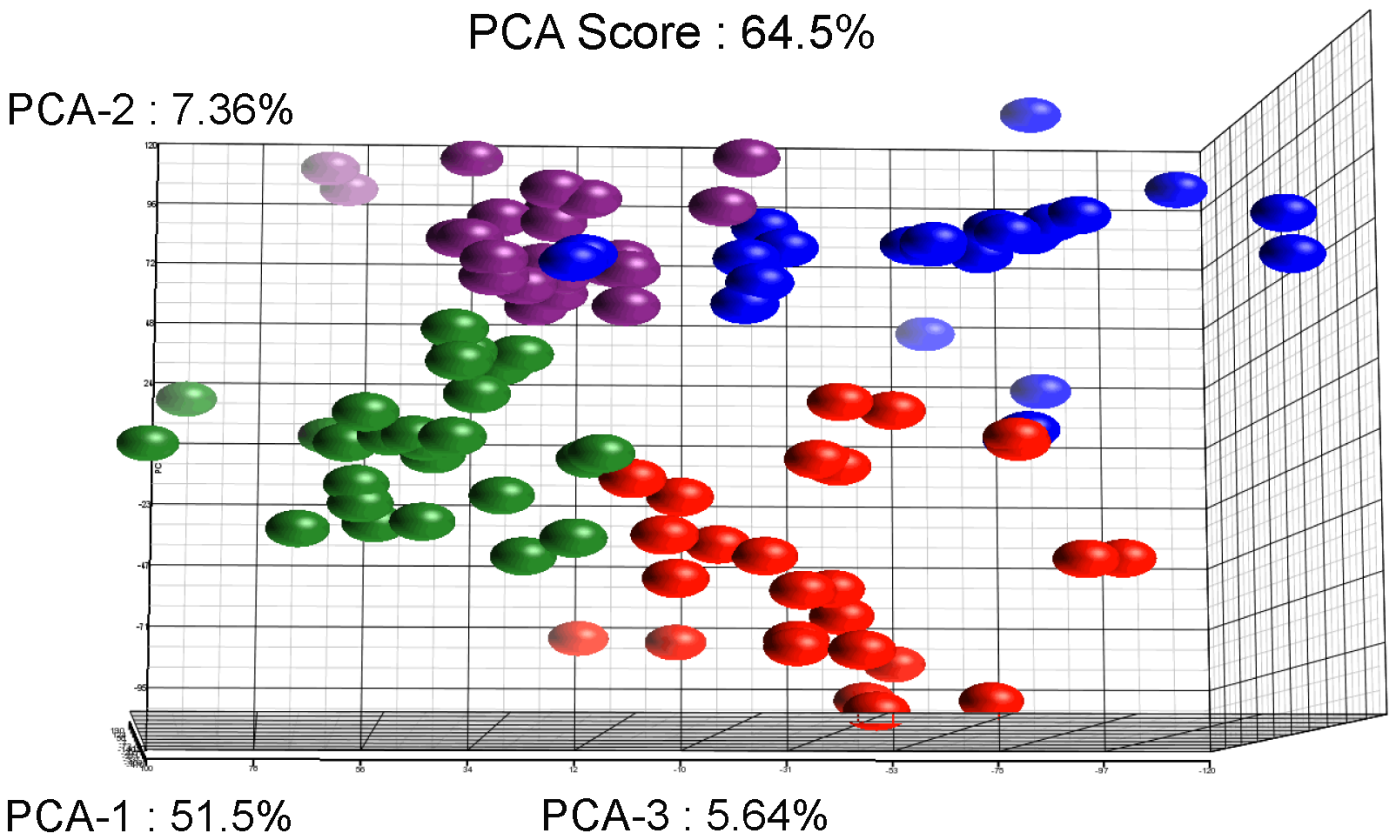
Results of the Principal Component Analysis. The figure represents the projection of the 48 arrays on the first three Principal Components. The sum of these principal three axes corresponds to 64.5% of the total variance. The representation corresponds to: The Rahman strain in contact with mucus (Red), Rahman strain in axenic culture (Blue), HMI:IMSS strain in contact with mucus (Green), and HMI:IMSS strain in axenic culture (Purple).

**Figure 4:**
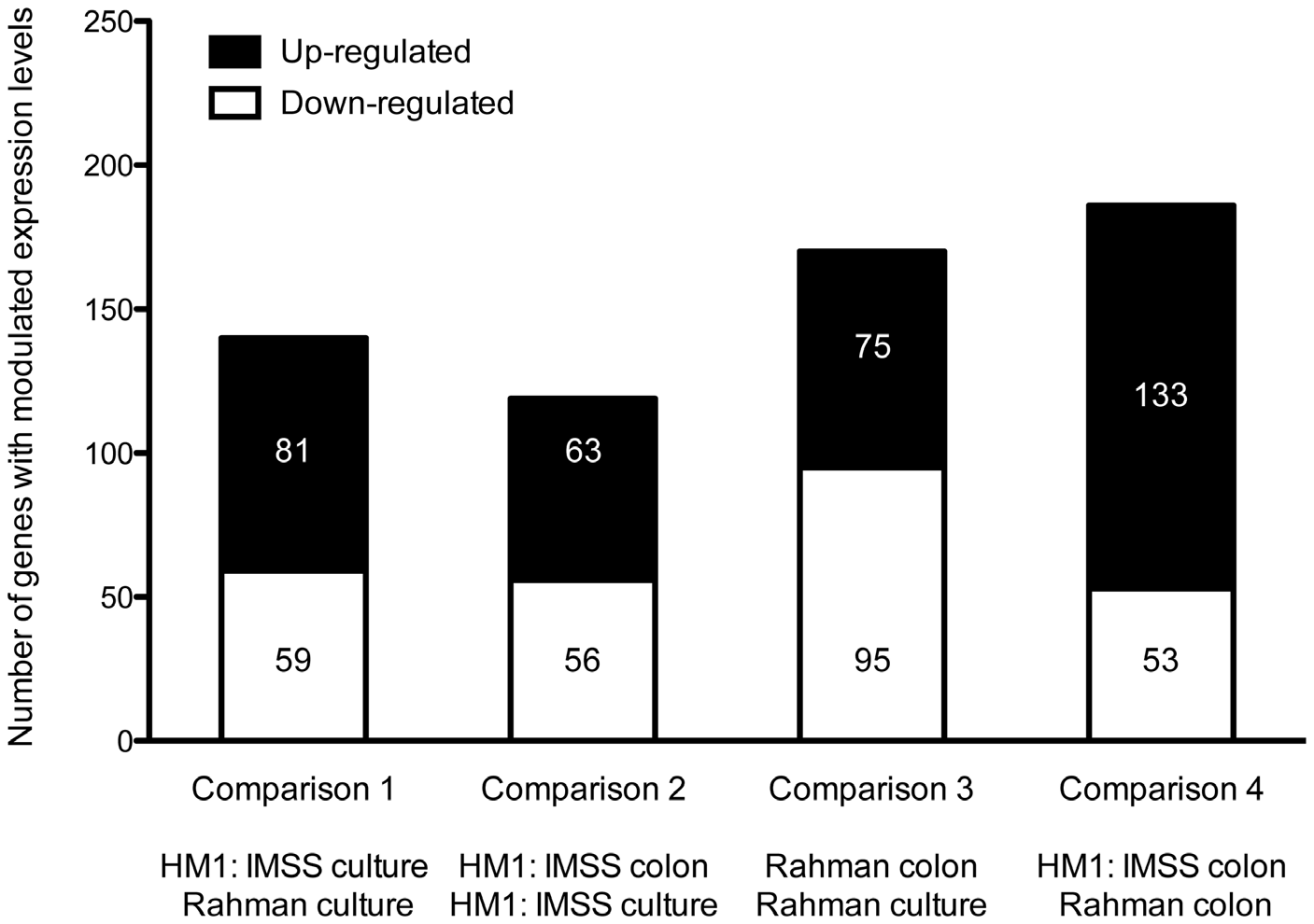
Summary of the microarray data. The transcriptomic modulations obtained in the 4 comparisons. In total, 614 transcripts with a fold change greater than two were significantly modulated (Bonferroni adjusted p-value<0.05).

Statistical evaluation by Principal Component Analysis (PCA) of the expression data showed that each comparison segregates as a distinct pool (Figure 3) indicating that (i) the biological replicates within each comparison showed similar gene expression patterns and (ii) the differences between the comparisons were higher than the individual variability, thereby validating our experimental settings. A stringent statistical threshold for the microarray data analysis was used, detecting a total of 614 genes with significantly modulated expression (Fold Change (FC) ≥2, Bonferroni adjusted p value≤0.05) (Figure 4). Eighty-one upregulated and 59 downregulated transcripts were different between HM1:IMSS and Rahman in axenic culture (Comparison 1) (Figure 2B, Table S3). Upon contact with the human colon explants (Comparison 2), 63 genes were upregulated and 56 were downregulated in HM1:IMSS, compared to axenic culture (Table S4). Comparison 3 indicates that 75 genes are upregulated and 95 downregulated in Rahman upon contact with the human colon explants, compared to axenic culture. Following mucus contact an additional 77 genes are upregulated in Rahman compared to HM1:IMSS (Table S5). Finally, the comparison between HM1:IMSS and Rahman upon contact with the human colon explants (Comparison 4), reveals 133 genes upregulated and 53 genes downregulated (Table S6). The 614 modulated genes were then manually classified into functional categories based on the gene annotation in AmoebaDB. These categories include, adhesion - cell surface molecules, translation - protein maturation, stress response, DNA-RNA regulation, cell signalling, nucleic acid metabolism, subcellular trafficking, oxidoreduction activities, proteolysis, carbohydrate metabolism, lipid metabolism, and cytoskeleton (Table 1).

**Table 1:** Classification of modulated transcripts into functional categories.

Comparison	1		2		3		4	
	HM1:IMSS culture/Rahman culture		HM1:IMSS colon/HM1:IMSS culture		Rahman colon/Rahman culture		HM1:IMSS colon/Rahman colon	
Functional categories	up	down	up	down	up	Down	up	down
Cell surface molecules	8	3	2	2	2	2	8	1
Protein synthesis	1	7	0	4	3	9	4	4
Stress	4	0	1	2	1	6	9	0
DNA-RNA regulation	2	1	10	1	6	8	8	3
Cell signalling	12	4	9	5	12	8	16	7
Nucleic acids metabolism	1	0	0	1	0	3	3	1
Aminoacids metabolism	0	0	0	7	2	5	0	0
Subcellular trafficking	0	0	1	0	1	0	3	0
Oxidoreduction	3	3	4	2	2	7	7	2
Proteolysis	3	4	2	2	4	1	4	4
Carbohydrate metabolism	10	0	4	1	3	1	11	1
Lipid metabolism	2	5	2	1	3	1	0	8
Cytoskeleton	2	4	1	6	0	5	3	2
Other	33	28	27	22	36	39	57	20
**Total**	**81**	**59**	**63**	**56**	**75**	**95**	**133**	**53**

Proteins were annotated according to Pathema and NCBI databases web sites.

### Expression profile of genes ubiquitously expressed in Rahman strain

Genes ubiquitously expressed in Rahman strain (n = 17, Table 2), were defined as those transcripts upregulated in both axenic culture and upon contact with human explants compared to that of HM1:IMSS. In particular, two α-1,3-mannosyltransferases (ALG2) and two Cysteine protease, CP-A8 and CP-A3 were included. CP-A3 has already been associated with non-virulent phenotypes as it is upregulated in the Rahman strain [11] and the non-virulent species, *E. dispar* [19]. Five genes encoding enzymes involved in lipid biogenesis were also present, including three lecithin:cholesterol acyltransferases that convert free cholesterol into cholesteryl ester, a START lipid binding domain containing protein, and a 1-O-acylceramide synthase. One gene belongs to the cytoskeleton functional category, coronin, as well as 2 genes encoding signalling molecules, were characteristic for the Rahman strain.

**Table 2:** The *E. histolytica* Rahman ubiquitous gene expression profile.

Gene ID	Description	Colon Rahman/HM1:IMSS		Culture Rahman/HM1:IMSS	
		FC	BF	FC	BF
**Translation-Protein Maturation**					
EHI_001990	alpha-1,3-mannosyltransferase ALG2	3,1	1,8E-24	3,6	3,4E-27
EHI_162230	alpha-1,3-mannosyltransferase ALG2	2,8	8E-25	2,9	7,5E-26
**DNA-RNA regulation**					
EHI_086540	replication factor C subunit 4	2,5	6,40E-21	2,2	7,80E-19
**Cell signalling**					
EHI_025360	14-3-3 protein 1	2,3	1,60E-16	2,4	5,20E-17
EHI_162340	serine/threonine protein kinase	5,4	9,20E-23	3,8	2,80E-18
**Proteolysis**					
EHI_159610	CP-A3	76,9	1,1E-35	27,8	2,1E-30
EHI_151400	CP-A8	2,3	6,5E-22	2,2	1,9E-20
EHI_169350	non-pathogenic pore-forming	2,6	3,40E-06	3,0	2,80E-08
EHI_199110	Lysozyme	2,4	1,70E-15	2,1	5,80E-13
**Lipid metabolism**					
EHI_025400	START domain containing protein	2,2	6,80E-19	2,3	2,90E-19
EHI_079060	1-O-acylceramide synthase precursor	5,5	2,20E-26	3,9	4,00E-22
EHI_035750	lecithin:cholesterol acyltransferase	2,4	8,10E-17	2,4	4,20E-17
EHI_065250	lecithin:cholesterol acyltransferase	34,5	1,30E-35	17,2	3,50E-31
EHI_099180	lecithin:cholesterol acyltransferase	10,9	9,90E-33	7,1	8,00E-29
**Other metabolisms**					
EHI_153060	acyl-CoA synthetase	4,7	5,20E-18	3,9	7,70E-16
EHI_153410	nitroreductase family protein	2,1	6,80E-18	2,2	2,70E-19
**Cytoskeleton**					
EHI_083590	Coronin	3,9	7,00E-14	2,6	1,30E-08

Genes showing increased expression in *E. histolytica* Rahman compared to HM1:IMSS both in culture and during contact with the human colon.

FC: Fold Change; BF: Bonferroni adjusted p value≤0.05.

### Gene expression profile specific to Rahman strain upon contact with the human colon explants

We identified 37 genes in Rahman trophozoites specifically upregulated only upon contact with the human colon explants (Table 3). The largest functional group is composed of factors involved in cell signalling such as several genes encoding phosphatases, kinases, a guanine exchange factor, a GTPase, and calcium binding protein 1 (CaBP1). The cysteine protease, CP-A4, is specific to this profile. Five genes encoding proteins that regulate lipid metabolism were found, including a gene encoding a long-chain-fatty-acid-CoA ligase (also called Fatty acyl-CoA synthetase) which catalyses the formation of fatty acyl-CoA, a substrate for β-oxidation and phospholipid biosynthesis [20] and another allele of lecithin:cholesterol acyltransferase. We also observed an increased expression of genes encoding proteins involved in DNA-RNA regulation, including a DNA repair and recombination protein, a DNA-directed RNA polymerase II, Piwi, a 5′-3′ exonuclease, and 1 RNA binding protein.

**Table 3:** The *E. histolytica* Rahman specific gene expression profile in contact with the mucus.

Gene ID	Description	Rahman colon/HM1:IMSS colon		Rahman colon/Rahman culture	
		FC	BF	FC	BF
**Adhesion-cell surface molecules**					
EHI_112490	serine-rich 25 kDa antigen protein	2,1	2,00E-09		
**Translation-Protein Maturation**					
EHI_170330	RIO1 family protein			2,91	1,40E-02
EHI_052400	translation elongation factor EF-1 alpha			2,05	1,30E-03
EHI_153840	midasin, putative	2,4	5,20E-12		
EHI_030610	HECT domain and RCC1-like protein			2,15	1,00E-14
**Stress**					
EHI_101120	heat shock protein 70, mitochondrial			2,12	1,30E-10
**DNA-RNA regulation**					
EHI_192830	DNA repair and recombination protein			2,12	1,70E-15
EHI_121760	DNA-directed RNA polymerase II			2,09	1,50E-06
EHI_125650	PIWI			2,19	9,90E-07
EHI_128220	5′-3′ exonuclease protein	2,0	9,90E-18		
EHI_153810	RNA recognition protein	2,9	2,00E-08	2,90	2,20E-08
**Cell signalling**					
EHI_017600	Ser/Thr protein phosphatase			2,04	2,40E-15
EHI_175490	dual specificity protein phosphatase	2,4	1,10E-13		
EHI_017600	Ser/Thr protein phosphatase			2,11	2,10E-17
EHI_015760	serine/threonine kinase	2,3	4,30E-21	2,22	2,40E-20
EHI_087800	tyrosine kinase			2,68	3,60E-20
EHI_034210	protein kinase	2,0	9,60E-12		
EHI_013170	protein kinase			2,37	2,20E-14
EHI_118810	protein kinase			2,06	4,80E-13
EHI_140330	protein kinase			2,82	1,80E-02
EHI_110980	RhoGEF	2,8	1,80E-16		
EHI_126270	Ras GTPase	2,0	8,20E-13		
EHI_164370	GTPase			2,21	9,60E-03
EHI_120900	calcium-binding protein 1 (EhCBP1)			2,64	1,70E-09
**Proteolysis**					
EHI_050570	CP-A4			2,19	3,80E-05
**Subcellular trafficking**					
EHI_045710	AP-2 complex protein			2,14	1,90E-06
**Oxidoreduction activities**					
EHI_026480	(2r)-phospho-3-sulfolactate synthase			2,41	3,10E-13
**Lipid metabolism**					
EHI_020250	lecithin:cholesterol acyltransferase	2,5	1,90E-17		
EHI_111000	fatty acid elongase	2,1	5,10E-11		
EHI_127830	long-chain-fatty-acid–CoA ligase	2,1	4,90E-19		
EHI_080220	Niemann-Pick C1 protein			2,16	1,30E-12
EHI_152280	serine palmitoyltransferase			2,02	9,60E-10
**Other metabolism**					
EHI_159710	alanine aminotransferase			2,80	1,30E-19
EHI_030180	alcohol dehydrogenase 3			2,52	1,30E-14
EHI_198760	alcohol dehydrogenase 3			2,49	1,10E-14
EHI_014030	NAD(P) transhydrogenase subunit alpha			2,00	6,60E-06
**Cytoskeleton**					
EHI_164430	actinin-like protein	2,3	6,10E-15		

Genes showing increased expression in Rahman after contact with the human mucus compared to Rahman in standard culture condition and HM1:IMSS in the same condition.

FC: Fold Change; BF: Bonferroni adjusted p value≤0.05.

### Gene expression profile in response to mucus contact common to both strains

Upon contact with the human colon explants, the response common to both *E. histolytica* strains, was defined by 13 genes (Table 4). The adhesion-cell surface molecules class includes the intermediate subunit 2 of the Gal/GalNAc lectin (Igl-2) and 1 newly identified protein containing a fibrinogen-binding domain (EHI_098440). Two cysteine protease-encoding genes were also identified for both strains as being upregulated. CP-A7 and an unannotated CP (EHI_010850) belonging to the peptidase C1A subfamily. Concerning energy metabolism, a gene implicated in lipid metabolism (long chain fatty acid CoA ligase) and 2 genes involved in carbohydrate metabolism were found (α-amylase and UDP-glucose 4-epimerase). A member of the Myb transcription factor family (EHI_008130) and several transcripts encoding signalling molecules were also found.

**Table 4:** Common gene expression profile in response to the mucus layer contact.

Gene ID	Description	HM1:IMSS colon/culture		Rahman colon/culture	
		FC	BF	FC	BF
**Adhesion-cell surface molecules**					
EHI_098440	Fibrinogen binding protein	2,4	1,4E-11	2,1	1,4E-09
EHI_183000	Igl2	7,6	1,50E-28	4,1	2,40E-21
**DNA-RNA regulation**					
EHI_008130	Myb protein, SHAQKYF	2,9	3,90E-13	2,1	1,00E-07
EHI_053170	RNA-binding protein	5,0	8,60E-12	4,0	1,60E-09
**Cell signalling**					
EHI_039850	Rho guanine exchange factor	2,2	7,10E-06	2,4	1,60E-07
EHI_109570	serine/threonine- protein phosphatase	2,1	2,60E-06	3,6	2,60E-14
EHI_136200	ser/thr protein phosphatase	2,0	7,40E-10	2,7	2,60E-15
EHI_164910	serine/threonine protein kinase	2,9	7,50E-21	3,5	6,00E-24
**Proteolysis**					
EHI_010850	Cysteine proteinase	2,1	2,00E-02	2,3	2,10E-03
EHI_039610	CP-A7	2,3	3,90E-03	2,4	2,30E-03
**Lipid metabolism**					
EHI_079300	Long-chain-fatty-acid–CoA ligase	5,7	5,80E-21	3,8	4,30E-16
**Carbohydrate metabolism**					
EHI_152880	alpha-amylase family protein	2,0	1,80E-14	3,2	2,50E-23
EHI_192050	UDP-glucose 4-epimerase	3,1	3,50E-14	3,1	3,80E-14

Genes showing increased expression in both *E. histolytica* HM1:IMSS and Rahman during contact with the human colon.

FC: Fold Change; BF: Bonferroni adjusted p value≤0.05.

### Expression profile of genes ubiquitously expressed in HM1:IMSS strain

The specific signature of HM1:IMSS in axenic culture and upon contact with the human colon explants is characterized by 39 transcripts (Table 5). This signature includes several surface associated proteins [21] namely the Gal/GalNAc lectin light subunits Lgl-1 and Lgl-5, the lysine- and glutamic acid- rich protein 1 (KERP1), the serine/threonine/isoleucine-rich protein (STIRP), and the cysteine protease CP-A5. The presence of CP-A5 is important to highlight since its activity is necessary for invasion of the human colon [5], [16]. The fact that we found well-known virulence factors associated with the HM1:IMSS gene signature confirms the relevance of the integrated analysis performed here.

**Table 5:** The *E. histolytica* HM1: IMSS ubiquitous gene expression profile.

		Colon HM1:IMSS/Rahman		Culture HM1:IMSS/Rahman	
Gene ID	Description	FC	BF	FC	BF
**Adhesion-cell surface molecules**					
EHI_035690	LGL-1	6,35	5,10E-30	5,40	3,40E-28
EHI_098210	KERP1	2,05	7,90E-10	3,04	4,70E-17
EHI_148790	LGL-5	16,23	6,50E-27	21,39	8,50E-29
EHI_004340	serine-threonine-isoleucine rich protein	2,21	1,20E-12	2,25	5,50E-13
EHI_012330	serine-threonine-isoleucine rich protein	2,70	1,40E-18	3,61	2,10E-23
EHI_004340	serine-threonine-isoleucine rich protein	6,74	8,10E-30	8,51	3,70E-32
EHI_025700	serine-threonine-isoleucine rich protein	7,35	2,20E-35	9,64	5,30E-38
**Translation-Protein Maturation**					
EHI_177660	isoleucyl-tRNA synthetase	2,17	2,60E-08	2,26	5,70E-09
**Stress**					
EHI_021780	heat shock protein 70	3,35	1,50E-19	2,61	2,20E-15
EHI_150770	heat shock protein 70	5,59	2,70E-28	5,16	2,40E-27
EHI_072140	heat shock protein 101	3,87	4,80E-19	3,78	2,40E-14
**DNA metabolism -RNA regulation**					
EHI_141830	RNA modification enzymes, MiaB-family	4,67	5,40E-28	4,00	6,80E-26
EHI_177540	thymidine kinase	2,52	8,60E-27	2,23	4,70E-24
**Cell Signalling**					
EHI_176700	AIG1 family protein	2,57	2,60E-17	2,45	2,00E-16
EHI_117470	Calmodulin	6,01	5,00E-26	7,16	6,90E-28
EHI_141840	Calmodulin	6,55	1,60E-21	6,22	5,10E-21
EHI_119910	Lipid phosphate phosphatase	3,53	5,30E-19	2,94	3,50E-16
EHI_177520	Rab X 11	2,03	5,70E-15	2,00	1,40E-14
EHI_177620	Calcium binding family protein	2,14	7,60E-14	2,08	3,40E-13
EHI_198330	Ras family GTPase	3,16	1,70E-09	3,05	4,40E-09
EHI_012490	ARF GTPase activating protein	2,99	8,70E-04	2,63	1,30E-02
**Oxidoreduction activities**					
EHI_138480	Iron-sulfur flavoprotein	4,23	2,30E-25	2,26	8,70E-15
**Proteolysis**					
EHI_008380	MP1-1	2,08	1,60E-17	2,17	1,20E-18
EHI_168240	CP-A5	3,61	5,90E-10	3,62	5,60E-10
EHI_179600	CP-C1-A peptidase domain	29,37	3,80E-25	22,73	1,30E-23
**Carbohydrate metabolism**					
EHI_000730	pyrophosphate-dependent phosphofructokinase	3,48	5,70E-19	2,39	1,00E-12
EHI_098570	fructose-1,6-bisphosphate aldolase	2,09	2,20E-08	2,08	2,60E-08
EHI_039190	aldose reductase	5,99	5,00E-26	3,72	4,40E-20
EHI_107560	aldose reductase	6,91	7,40E-25	3,31	4,80E-18
EHI_157010	aldose reductase	4,07	1,10E-19	2,58	8,80E-13
EHI_118440	beta-amylase	3,40	6,00E-16	2,63	5,40E-12
EHI_192590	beta-amylase	16,94	5,30E-31	25,08	1,20E-33
EHI_088020	alcohol dehydrogenase	5,18	2,40E-26	2,95	2,30E-18
EHI_160670	alcohol dehydrogenase 3	4,45	8,10E-21	3,33	7,30E-17
**Cytoskeleton and miscellaneous**					
EHI_158570	Actobindin	2,37	1,40E-09	2,19	3,20E-08
EHI_163260	Copine	2,38	8,30E-23	2,05	3,50E-19
EHI_069060	LIM zinc finger domain containing protein	3,33	1,60E-14	2,04	1,20E-06
EHI_188600	dentin sialophospho protein precursor	3,60	1,00E-07	2,51	8,00E-04
EHI_015120	leucine rich repeat protein, BspA family	2,31	1,10E-07	2,12	2,70E-06

Genes showing increased expression in *E. histolytica* HM1:IMSS compared to Rahman both in culture and during contact with the human colon.

FC: Fold Change; BF: Bonferroni adjusted p value≤0.05.

Genes encoding proteins were identified to be important for the amoebic stress response and include heat shock proteins-70 (HSP-70) and HSP-101, a calcium binding protein involved in signalling, two calmodulins, and several GTPases from the AIG protein family. Three proteases-encoding genes also characterized the gene expression profile specific for the HM1:IMSS strain, namely an unannotated Cysteine protease containing a C1-A peptidase domain and a metalloprotease MP-1. Several genes implicated in carbohydrate metabolism, including 5 genes encoding glycolytic enzymes, phosphofructokinase, fructose 1–6 aldolase, and aldose reductase, and two genes encoding β-amylase were found.

### Gene expression profile specific to HM1:IMSS strain upon contact with the human colon explants

HM1:IMSS trophozoites specifically upregulate 40 genes upon contact with human colon explants (Table 6 and 7) and two points are worth to notice in particular. First, it is the upregulation of 6 genes encoding proteins annotated as regulators of nonsense transcripts. They all contain a RNA helicase domain belonging to the super family 1 (SF1). This RNA helicase domain promotes structural transitions of RNA or RNA-protein complexes. We further found Myb 13 (EHI_053000) that belongs to the MybR2R3 family of transcription factors and which has been reported to bind a DNA consensus Myb recognition element *in vitro* [22]. Second, it is the upregulation of proteins involved in signalling, including a phosphatase, a kinase, 2 Rab GTPases, 3 Ras GTPases, and a cyclin. Genes linked to the stress response were identified and include 2 HSP-70 genes and 2 ubiquitin genes. Furthermore, the 2 genes implicated in sugar catabolism were also upregulated and they encode a starch binding protein (EHI_074010) and another allele of β-amylase (EHI_035700) respectively.

**Table 6:** The *E. histolytica* HM1:IMSS specific gene expression profile in contact with the mucus.

Gene ID	Description	HM1:IMSS colon/Rahman colon		HM1:IMSS colon/HM1:IMSS culture	
		FC	BF	FC	BF
**Adhesion- surface molecules**					
EHI_073630	serine-threonine-isoleucine rich protein	2,5	2,70E-22		
**Translation-Protein Maturation**					
EHI_053830	Ribonucleoprotein	2,0	1,60E-10		
EHI_006170	eukaryotic translation initiation factor 6	2,1	5,60E-08		
EHI_126920	asparaginyl-tRNA synthetase	2,2	2,20E-10		
**Stress and Proteolysis**					
EHI_052860	heat shock protein 70, putative	2,0	1,20E-18		
EHI_065320	heat shock protein 70, putative	2,2	8,80E-05		
EHI_083410	Ubiquitin	3,2	2,00E-13	2,2	1,10E-07
EHI_178340	Ubiquitin	2,1	2,60E-04		
EHI_078710	probable proteasome subunit beta type 2	2,0	6,30E-14		
**DNA-RNA regulation**					
EHI_053000	Myb 13			2,2	1,80E-09
EHI_193520	regulator of nonsense transcripts			2,6	1,20E-11
EHI_178520	regulator of nonsense transcripts	8,6	1,00E-18	2,2	1,30E-12
EHI_070810	regulator of nonsense transcripts	7,4	1,30E-23	9,1	1,60E-25
EHI_110840	regulator of nonsense transcripts	4,5	3,50E-23	4,7	6,80E-24
EHI_148970	regulator of nonsense transcripts	3,9	9,40E-18	3,7	3,20E-21
EHI_043440	regulator of nonsense transcripts	6,4	6,00E-23		
EHI_131080	DEAD/DEAH box helicase	2,4	4,40E-13	2,5	3,10E-13

Genes showing increased expression in HM1:IMSS after contact with the human mucus compared to HM1:IMSS in standard culture condition and Rahman in the same condition.

FC: Fold Change; BF: Bonferroni adjusted p value≤0.05.

**Table 7:** The *E. histolytica* HM1:IMSS specific gene expression profile in contact with the mucus.

Gene ID	Description	HM1:IMSS colon/Rahman colon		HM1:IMSS colon/HM1:IMSS culture	
		FC	BF	FC	BF
**Cell signalling**					
EHI_056420	protein phosphatase			2,1	1,90E-12
EHI_012220	protein kinase domain containing protein	2,2	6,40E-18	2,7	8,40E-23
EHI_146510	Rab 1-B	2,0	1,50E-15		
EHI_164900	Rab 2-D	2,1	3,50E-13		
EHI_124610	Ras family GTPase	2,9	5,60E-09		
EHI_058520	Ras family GTPase	3,0	2,20E-03	2,7	1,40E-02
EHI_141430	Ras family GTPase	2,0	1,60E-13		
EHI_121830	cyclin family protein			2,1	6,10E-19
**Nucleic acids metabolism**					
EHI_047750	Nucleotide-binding protein	2,7	2,80E-14		
**Subcellular trafficking**					
EHI_069480	HEAT repeat domain containing protein	2,3	1,30E-12		
EHI_073420	Dopey domain protein	2,0	6,10E-10	3,0	4,20E-17
EHI_095820	ATP-binding cassette protein	2,2	1,10E-12		
EHI_095820	ABC transporter,	2,3	1,20E-15		
**Oxidoreduction activities**					
EHI_197340	Sulfotransferase	2,7	6,20E-09	2,5	1,70E-07
EHI_049620	Fe-S cluster assembly protein NifU	2,2	4,80E-06	2,3	9,50E-07
EHI_067950	rodhanase-like domain containing protein			3,4	1,80E-23
**Amino acids metabolism**					
EHI_004920	S-adenosylmethionine synthetase			2,0	9,70E-05
**Carbohydrate metabolism**					
EHI_074010	starch binding protein	2,1	1,30E-10		
EHI_035700	beta-amylase	2,5	7,40E-12		
**Cytoskeleton and miscellaneous**					
EHI_052740	dynamin-like protein	2,4	1,40E-09	2,7	4,40E-11
EHI_161300	leucine rich repeat protein, BspA family	2,1	2,10E-11		
EHI_134960	Skp1 family protein	2,0	6,80E-10		
EHI_057670	20 kDa antigen	2,2	7,30E-10		

Genes showing increased expression in HM1:IMSS after contact with the human mucus compared to HM1:IMSS in standard culture condition and Rahman in the same condition.

FC: Fold Change; BF: Bonferroni adjusted p value≤0.05.

### Global transcriptome landscape of virulent and non-virulent strains

**Figure 5:**
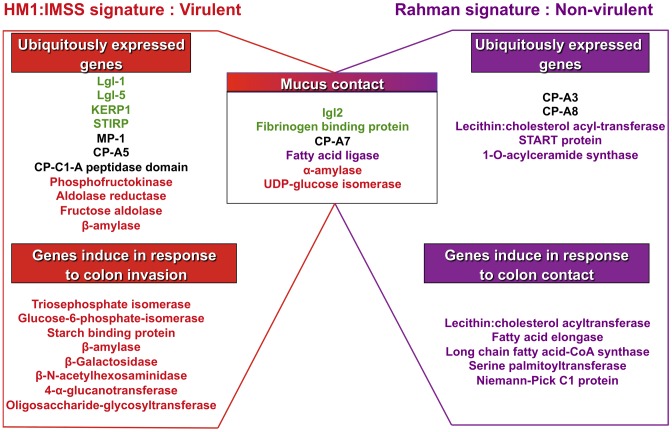
Transcriptomic landscape describing the virulent and non-virulent signatures of *E. histolytica*. The scheme summarizes the transcriptomic map of *E. histolytica* highlighting two categories of genes: those permanently expressed in each strain and those overexpressed when amoebas are in contact with human colon explants. The signature of HM1:IMSS is on the left and the Rahman non-virulent signature is on the right. Central in the scheme are the genes common to the two strains and only upregulated when the parasites are in contact with the mucus. The adhesion-cell surface molecules are depicted in green; proteases in black; carbohydrates metabolism in red; lipid metabolism in purple. Only the most striking differences were highlight in this figure, others categories are described in tables 2, 3, 4, 5, and 6.

The 5 profiles established above were combined to depict the transcriptomic landscape associated with HM1:IMSS (virulent and intestinal invasive) and the Rahman strain (non-virulent and intestinal non-invasive) phenotypes of *E. histolytica*. We highlighted in Figure 5 the well-known virulent factors and the metabolic pathways herewith identified. The specific signature for the mucus-invading HM1:IMSS strain is composed of the following 3 profiles: the HM1:IMSS ubiquitously expressed genes (common to culture and mucus), the common gene expression profile of both strains in response to mucus contact, and the gene expression profile induce in response to colon invasion (exclusive to HM1:IMSS and inherent to mucus invasion). Thus the virulent phenotype of *E. histolytica* associated with HM1:IMSS is characterized by the expression of genes involved in adhesion (Lgl-1, Lgl-5, Igl-2, KERP1, STIRP, putative fibrinogen binding protein), proteolytic activities (MP-1, CP-A5, CP), and carbohydrate metabolism (phosphofructokinase, aldose reductase, fructose aldolase, β-amylase, α-amylase, UDP-glucose isomerase, triosephosphate isomerase, glucose-4-epimerase, 4-**α**-glucanotransferase, and oligosaccharide-glycosyltransferase).

The specific signature associated to the non-virulent Rahman strain consists of the ubiquitously expressed gene profile (common to culture and mucus) in addition to the common gene expression profile in response to mucus contact and the gene expression profile specifically induce in Rahman in response to colon contact (exclusive to Rahman and inherent to mucus contact). Thus the non-virulent phenotype of *E. histolytica* associated to Rahman strain is characterized by the independence from adhesion molecules, the activation of genes encoding proteases activities (CP-A3 and CP-A8) distinct from virulent trophozoites, and the importance of lipid metabolism (lecithin: cholesterol acyl-transferase, START protein, 1-O-acylceramide synthase, fatty acid elongase, long chain fatty acid-CoA synthase, serine palmitoytransferase, and Niemann-Pick C1 protein). Since the Rahman strain expresses this particular set of genes, we conclude that it does not favour colonic mucosa invasion.

### Carbohydrate metabolism genes are upregulated in virulent strain

A striking result of this study is the discovery of specific distinction concerning energy metabolism pathways activated by non-virulent and virulent strains when they are in contact with the mucus layer. Rahman strain is characterized by an increased expression of genes related to lipid metabolism (Table 2 and 3), whereas HM1:IMSS strain is characterized by upregulation of genes encoding proteins involved in carbohydrate metabolism (Table 5 and 7). To test for functional enrichment in genes upregulated in HM-1:IMSS versus Rahman strains during colon invasion, we performed a hyper-geometric test for gene ontology enrichment [23] and gene set enrichment analysis [24] for KEGG pathway [25]. In the hyper-geometric test, carbohydrate catabolic process (GO:0016052), among other carbohydrate metabolism related gene ontology terms, was significantly enriched (Table S7). Moreover, in gene set enrichment analysis for KEGG pathway, Glycolysis/Gluconeogenesis (ehi00010) and, Fructose and mannose metabolism (ehi00051) were also significantly enriched (Table S8).

**Figure 6:**
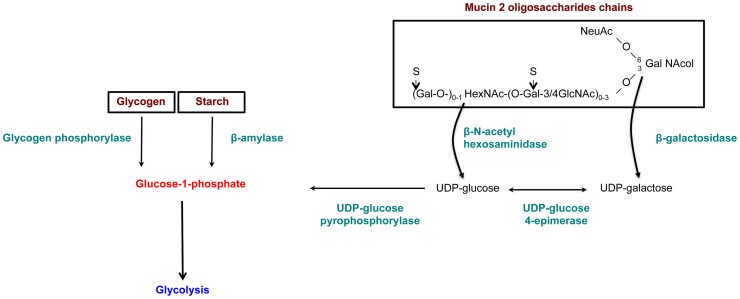
Enzymes overexpressed and involved in carbohydrate metabolism specific to the virulent HM1:IMSS strain. Genes involved in glycan metabolism with significant and specific overexpression in HM1:IMSS are shown in green. The genes were chosen according to a Bonferroni adjusted p value≤0.05 and without fold change cutoff (Table S9).

The results from gene enrichment tests prompt us to take a closer look at the carbohydrate metabolism genes that are significantly upregulated in the HM1:IMSS strain (without fold-change cut-off), 39 additional genes involved in carbohydrate metabolism were found and listed in Table S9. In particular we identified genes encoding enzymes that are potentially involved in carbohydrate retrieval from MUC2: β-galactosidase (EHI_170020) and β-N-acetylhexosaminidase (EHI_148130) and 3 genes involved in the production of glucose-1-phosphate - glycogen phosphorylase (EHI_110120), 2 other alleles of β-amylase (EHI_098200, EHI_148800), and UDP-glucose pyrophosphorylase (EHI_000440). Glycogen phosphorylase catalyses the rate-limiting step in glycogen degradation by releasing glucose-1-phosphate from the terminal α-1,4-glycosidic bond, β-amylase releases maltose from the polysaccharide chain by hydrolysis of α-1,4-glucan linkages, and UDP-glucose pyrophosphorylase that catalyses the formation of glucose-1-phosphate and UDP from UDP-glucose. In addition, among the 11 enzymes involved in the glycolytic pathway, 7 were specifically induced in the HM1:IMSS strain: phosphoglucomutase, aldose reductase, glucose-6-phosphate isomerase, phosphofructokinase, fructose-1,6-biphosphate aldolase, triosephosphate isomerise, and phosphoglycerate mutase (Table S9 and Table 5). A global view of potential activities of these metabolic enzymes accounting for MUC2 degradation by HM1:IMSS during the invasive process is presented in Figure 6.

### Reduction of *E. histolytica* β-amylase levels by dsRNA interference decrease the depletion of the mucus layer

**Figure 7:**
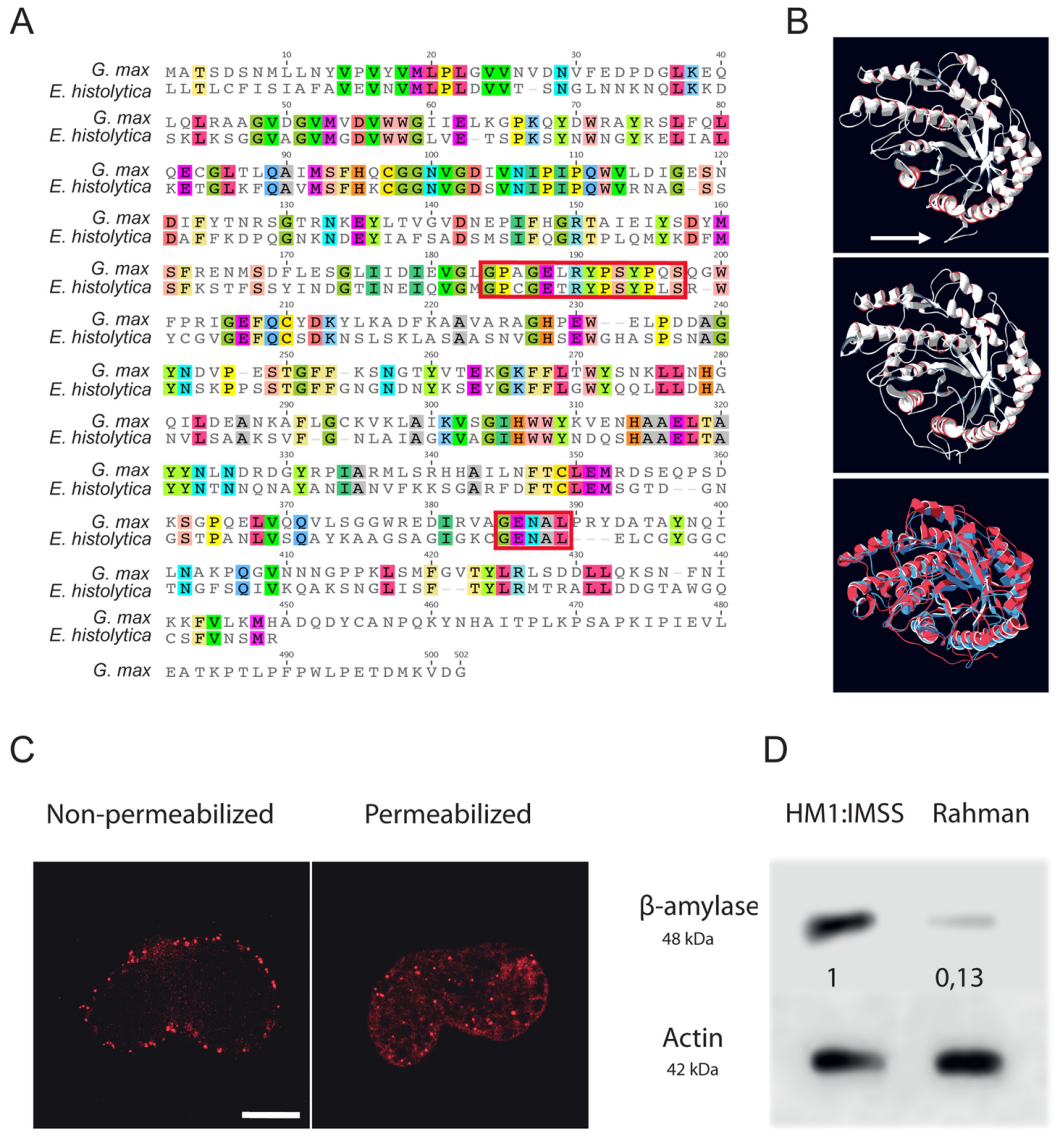
Functional characterization of *E. histolytica* β-amylase. A. Amino acid sequence alignment of full-length β-amylase homologues in *Entamoeba histolytica* (EHI_192590) and *Glycine max* (BMY1: 547931). Identical residues are highlighted. The red boxes indicate the two conserved catalytic domains containing two glutamic acids (E185 and E378) involved in the catalytic reaction. B. Bioinformatic structural model of β-amylase in *E. histolytica* predicted using LOMETS software. A 3D structural search of *E. histolytica* β-amylase match β-amylase from *G. max* as the best-hit template. The upper panel shows the predicted structural model of *E. histolytica* β-amylase. The middle panel represents the crystal structure of *G. max* β-amylase [26] and the lower panel shows the merge between the two structures. Note (i) the strong structural homology between the two enzymes and (ii) the N-terminal tail of *E. histolytica* β-amylase, which was predicted as a transmembrane domain (white arrow). C. Cellular localization of *E. histolytica* β-amylase. Trophozoites were fixed and labelled by immunofluorescence for β-amylase. Confocal microscopy image analysis revealed a cell surface localization in non-permeabilized trophozoites, and in cytoplasmic dots in permeabilized parasites. Scale bar = 5 µm. D. Immunodetection of β-amylase (48 kDa) in crude extracts of HM1:IMSS or Rahman strains. 30 µg of proteins were loaded and resolved on a 12% SDS-PAGE gel. Proteins were transferred onto PVDF membranes and probed with an anti β-amylase specific antibody. Actin was used as a loading control. Rahman strain synthetizes roughly 13% of β-amylase compared to HM1:IMSS taken as 100%.

Based on the sharp increase in β-amylase transcript level (EHI_192590, fold change up to 25) in HM1:IMSS strain and the enrichment of carbohydrate metabolism genes, we opted to further investigate the role of β-amylase during human mucus invasion. The predicted 3D structure of *E. histolytica* β-amylase using LOMETS software [26] reveals a strong structural homology to the crystal structure of *Glycine max* β-amylase. Analysis of the β-amylase amino acid sequence by BLAST reveals similarity (42% pairwise identity, E value = 9e^−119^) with β-amylase of *G. max*. Importantly, two glutamic acids residues (E185 and E378) involved in the catalytic activity are present at the homologous position in the *E. histolytica* enzyme (Figure 7A). An additional trans-membrane domain was predicted for *E. histolytica* β-amylase (Figure 7B). Based on significant protein homologies with β-amylase from plants, we took advantage of an existing antibody against β-amylase, which recognizes these enzymes. The specificity of this commercial antibody was confirmed by expressing the amoebic β-amylase encoding gene (EHI_192590) in bacteria and western blot analysis (Figure S1). We observed that the protein is localized both on the cell surface and at focused locations in cytoplasm by using immunofluorescence on trophozoites (Figure 7 C).

**Figure 8:**
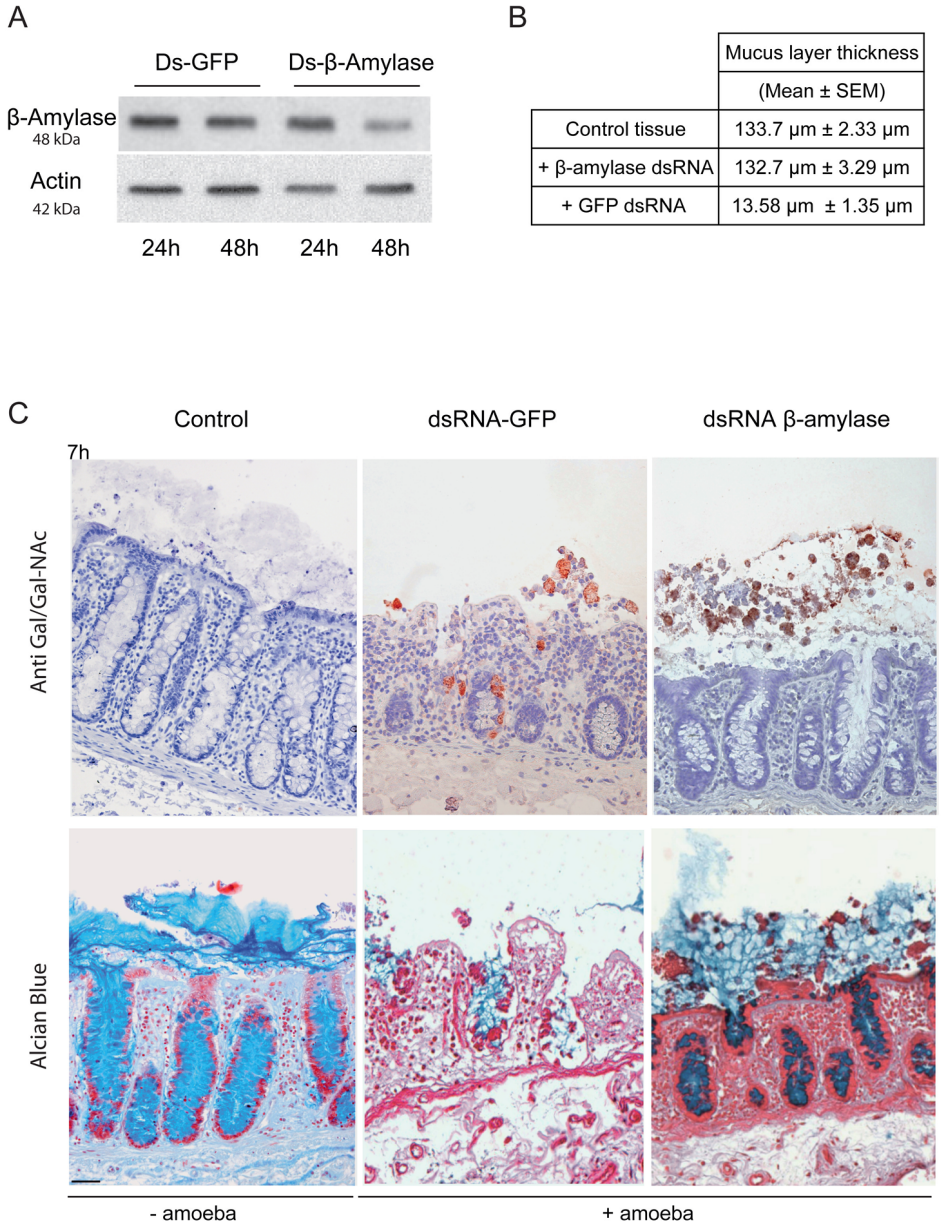
Depletion of ß-amylase in HM1:IMSS trophozoites prevent mucus layer degradation. A. Immuno-detection of β-amylase in dsRNA treated parasites. Trophozoites were treated for 24 or 48 h with control dsRNA or β-amylase specific dsRNA. Crude extracts were analysed by western blotting. Protein loading was normalized with respect to actin. After 48 h, β-amylase amounts were reduced by 75.5% (SEM ± 4.6%; n = 3) in comparison to the control. B. Quantification of the mucus layer degradation. After 7 h of incubation with control dsRNA treated trophozoites or β-amylase specific dsRNA treated trophozoites colonic explant were fixed and stained with Alcian blue to visualize the mucus layer. In the presence of β-amylase specific dsRNA treated trophozoites the thickness of the mucus layer is not altered as compared to the control tissue (132.7 µm vs 133.7 µm) while in the presence of GFP dsRNA treated trophozoites the mucus layer thickness decrease to 13.58 µm. C. Histological study of mucosal invasion. Trophozoites treated for 48 h with control dsRNA or β-amylase specific dsRNA were incubated with the human colon explant and tissue section were analysed as described in Figure 1. Decrease in β-amylase abundance inhibits mucosa invasion after 7 h of incubation. β-amylase deficient trophozoites were still associated with the mucus layer while control-treated trophozoites have depleted the mucus layer and invaded the *lamina propria*. Scale bar = 50 µm.

*Entamoeba histolytica* possesses 8 copies of the β-amylase encoding gene (EHI_009020, EHI_035700, EHI_049700, EHI_148800, EHI_058340, EHI_118440, EHI_192590, EHI_098200) whose protein lengths range from 436 to 444 amino acids. We confirmed this information by taking advantage of RNA-Seq analysis recently performed in our laboratory [27] that the most highly expressed β-amylase genes in HM1:IMSS were EHI_192590, EHI_098200, and EHI118440 (Figure S2). In our microarray experiments, EHI_192590 is the most upregulated compared to Rahman (mucus and culture conditions) and in addition EHI_035700 is only overexpressed in HM1:IMSS during colon invasion. Levels of expression were very low in the Rahman strain and we confirmed by western blot that β-amylases were indeed present in cultured HM1:IMSS strain and highly reduced in Rahman (7.7 fold decrease at the protein level, Figure 7D). In order to gain insights into the role of β-amylase during mucus invasion, we knock down the expression of β-amylase in the HM1:IMSS strain using a dsRNA-based RNA interference approach [28]. We designed a specific dsRNA targeting the transcripts of all 8 copies (see material and methods). Total protein extracts were analysed using western blot after 24 h and 48 h of incubation with the specific β-amylase dsRNA or a control dsRNA (i.e GFP dsRNA). After 48 h of incubation, the β-amylase quantity was decreased by 75.5% (SEM ± 4.6%; n = 3) in comparison to the control (GFP dsRNA-treated trophozoites) without impacting the growth of theses trophozoites (Figure 8A). The viability of dsRNA treated trophozoites was determined upon an hour incubation in Krebs buffer by trypan bleu exclusion test (percentage of cell death was for dsGFP = 11.2±3.4 sd, and for ds β-amylase = 10.8±2.9 sd., n = 3). HM1:IMSS trophozoites with reduced levels of β-amylase were then challenged for human colon invasion. We observed by histological analysis that after 7 h of incubation, tissue invasion by β-amylase dsRNA-treated trophozoites was abolished, while these trophozoites were still associated with the mucus layer (Figure 8B and 8C). In contrast, GFP dsRNA treated parasites (used as a control) depleted the mucus layer and penetrated the *lamina propria*, as wild-type HMI:IMSS trophozoites. Measurement of the mucus thickness after 7 h incubation in the presence of β-amylase dsRNA-treated trophozoites (133.7 µm SEM ± 2.33 µm) was comparable to the mucus thickness of the tissue control incubated without trophozoites (132.7 µm SEM ± 3.29 µm). However, in the presence of GFP dsRNA treated parasites the mean thickness of the mucus layer was significantly decreased to 13.58 µm (SEM ± 1.35 µm, p<0.0001).

## Discussion

*E. histolytica* colonizes the human gut mainly as a parasite. Only 1 in 5 infections leads to disease [29]. The classical view of amoebic infection outcome is that the virulence of *E. histolytica* is the consequence of the interactions between host, parasite, and environmental factors. Although the evidence supporting the phenotypic conversion of a strain from non-virulent to virulent is currently lacking, it is admitted that a latent period between infection and disease is due to parasite adaptation to the host via modifications in gene expression [30]. However, *E. histolytica* strains isolated from healthy asymptomatic carriers do not reproduce infection in animals implying that there is an unidentified mechanism regulating gene expression in addition to adaptation. Using the human colon explant model [5], we compared the transcriptome modulation upon mucus contact of *E. histolytica* strains isolated from asymptomatic (Rahman) or symptomatic (HM1:IMSS) patients. Notice that only one representative virulent strain (HM1-IMSS) and only one representative non-virulent strain (Rahman) were compared in this study. Trophozoites from these isolates has been in culture for decades and likely may harbour differences unrelated to virulence, however these represent the best characterized amoebic isolates from genomics and biological point of views. Indeed, non-virulent Rahman trophozoites bound to the mucus but neither depleted the protective barrier nor invaded and destroyed the tissue, in contrast to HM1:IMSS virulent trophozoites. The transcriptome analysis identified genes: (i) ubiquitously expressed in each strain, (ii) common to the 2 strains interacting with human mucus, and (iii) specifically expressed in response to colon contact. The transcriptome of amoebae able to invade the mucus was characterized by several virulence factors (the Gal/GalNAc lectin, STIRP, KERP1 and CP-A5) already described for their participation in the pathological process or over-expressed in virulent amoebic strains [21]. Also identified were proteins such as the SHAQKYF (Myb 13) transcription factor regulating the expression of genes related to signal transduction, vesicular transport, heat shock response and virulence [22] as well as transcripts linked to stress responses and to signalling pathways, including the GTPase AIG1 known to be expressed during colonization of the mouse intestine [31] and in pathogenic *E. histolytica* [32].

**Figure 9:**
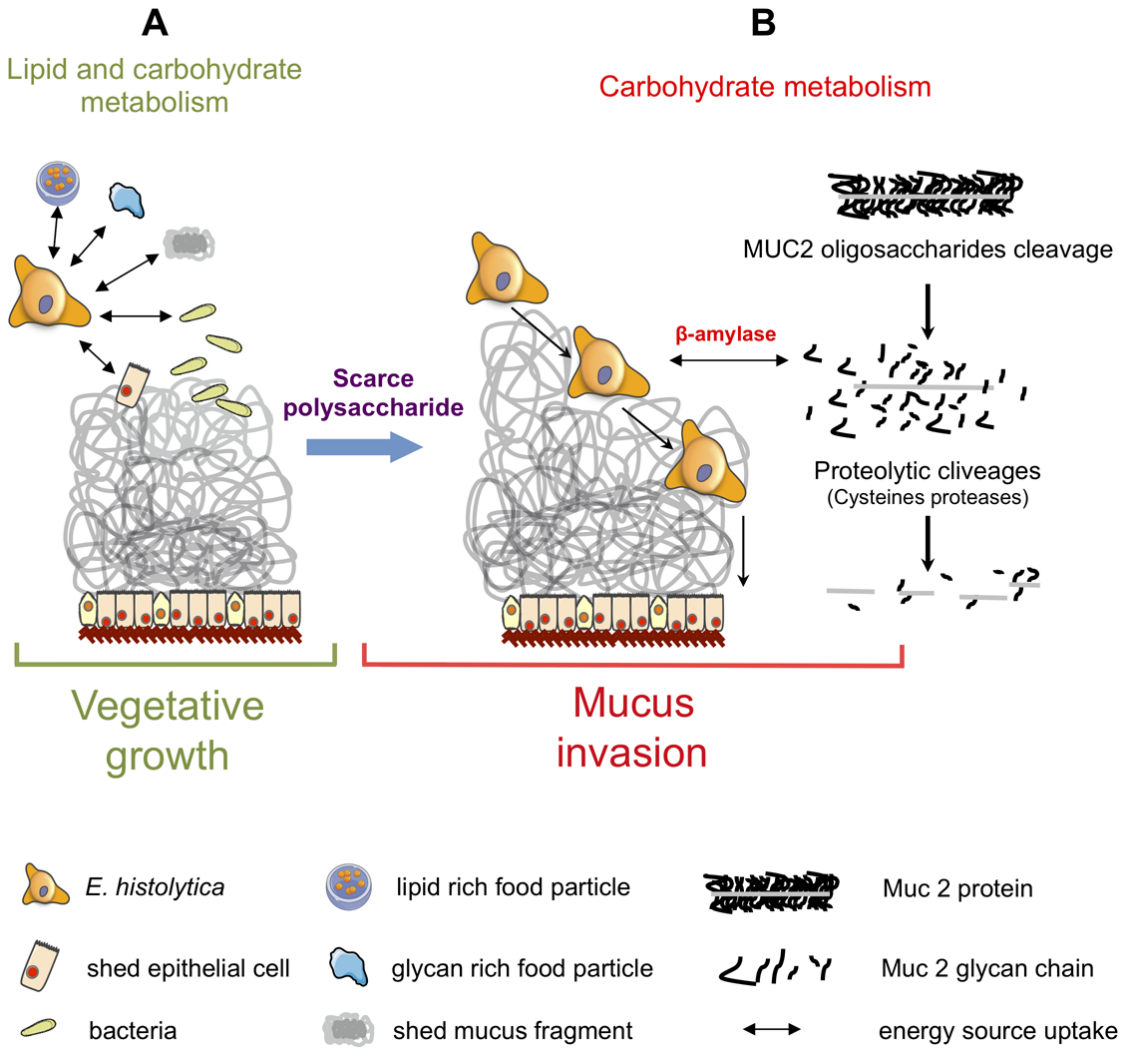
Schematic of *Entamoeba histolytica* activities leading to mucus layer depletion and invasion of the human colon. During its vegetative life style (A), *E. histolytica* exploits lipids (from lipid-rich food particles, bacteria, and shed epithelial cells) and carbohydrates (undigested glycan-rich food particles provided by the bolus or shed mucus fragment) present in the colonic environment. (B) When dietary polysaccharides are scarce, we hypothesize that *E. histolytica* turns to host mucus by first deploying a set of polysaccharide hydrolases that depletes the protective oligosaccharide side chains of mucin which can be then targeted by cysteine proteases leading to the depletion of the protective mucus barrier and allowing subsequent invasion of the mucosa. This adaptive foraging could reflect the coevolved functional versatility of *E. histolytica* glycobiome and the structural diversity of host mucus glycans involved in the interaction.

Besides the involvement of the above cited virulence factors, the remarkable feature of colon explant invasion concerns the changes in expression of genes encoding enzymes involved in the carbohydrate metabolism. In addition to several enzymes implicated in the production of glucose-1-phosphate, upregulation of genes encoding the majority of enzymes involved in glycolysis was characteristic to mucus depletion. Therefore, we hypothesized that carbohydrate metabolism might play a role in sustaining the invasive behaviour of the virulent strain during intestinal invasion. Indeed when accessibility of polysaccharides in the lumen is decreased and glucose levels are low, virulent *E. histolytica* might be able to adapts its transcriptome to proficiently utilized host mucus glycans as its carbon source. Here we proposed a sequential mode of MUC2 degradation, involving the release of oligosaccharides from MUC2 by glycosidases (e.g. beta-galactosidase and beta-N-acetylhexosaminidase, upregulated in virulent strain during colon invasion), and followed by cleavage of the exposed protein backbone by proteases (Figure 6 and Figure 9). We speculate β-amylase might play a role in breaking down the already released oligosaccharides into sugars as carbon sources for energy production. Thus, the reduced β-amylase activity in the dsRNA treated strain might hamper the utilization of MUC2 as the carbon source for glycolysis. The upregulation of multiple genes in the glycolytic pathway in the virulent strain during colon invasion correlates with this speculation and we interpret the upregulation of these genes in the virulent strain as the consequence of utilization of MUC2 as the carbon source. This hypothesis supports our previous findings showing that *E. histolytica* virulence increased when in the presence of a low glucose environment [33]. This scenario also fits well with previous findings indicating that *E. histolytica* depletes colonic mucin oligosaccharide side chains by using a glycosidase activity [34]. Following the breakdown of MUC2 oligosaccharides, the protein backbone is no longer protected and may be degraded by specific amoebic proteases as has been previously demonstrated [35], [36].

In this work we highlighted β-amylase, because it is a protein absent from the mammalian kingdom proteome and is strongly overexpressed (25-fold) in HM1:IMSS strain. The enzyme β-amylase acts on the α-1,4 glycosidic bonds and catalyses the breakdown of starch into maltose (a glucose dimer). Using a dsRNA-based strategy, we decreased β-amylase protein levels in HM1:IMSS strain, and resulted in reduced mucus layer depletion and mucosa invasion. The β-amylase activity and its substrate in the invasive process have yet to be determined. The fact that β-amylase does not exist in the human genome makes this enzyme a potential therapeutic target to inhibit amoebic intestinal invasion.

*Entamoeba histolytica* typically feeds on bacteria in the intestinal lumen. Microbial inhabitants of the gut, which can also have an influence on metabolic processes, such as energy extraction from food and host mucus glycan, can be considered as an environmental factor that contributes to amoebic maintenance in the colon lumen and further in the pathology. Our hypothesis (Figure 9) is in line with findings obtained from bacteria resident in the mucus layer, in which they are capable to adapt their gene expression to gut diet content. For example gene expression profiling of *Bacteroides thetaiotaomicron*, revealed that rich polysaccharide diets are associated with a selective upregulation of glycoside hydrolases (e.g. xylanases, arabinosidases, and pectate lyase). These bacteria also upregulate genes encoding enzymes involved in delivering glucose to the glycolytic pathway [3]. When these bacteria are in the presence of a unique glucose diet devoid of polysaccharides, the induction of a different subset of glycoside hydrolases is activated including enzymes necessary for retrieving carbohydrates from mucus glycans, as well as enzymes that increase accessibility to host glycans [3]. We proposed that the ability of virulent *E. histolytica* trophozoites to exploit carbohydrate resources derived from the human mucus might be one of the factors powering intestinal amoebiasis.

## Materials and Methods

### Ethics statement

Healthy segments of human colon were obtained from patients undergoing colon surgery. Patient-written informed consent was obtained at Foch Hospital and the data were analysed anonymously at the Pasteur Institute. Tissues were processed according to the French Government guidelines for research on human tissues and the French Bioethics Act, with the authorization from the “comité de protection des personnes, Ile de France VII” and the “Institut Pasteur Recherche Biomedicale” investigational review board (RBM./2009.50).

### Bacterial strain, cells, and culture condition

RNAseIII-deficient *Escherichia coli* strain HT115 (rnc14::ΔTn10) was grown in LB-broth containing ampicillin (100 µg/ml) and tetracycline (10 µg/ml). *Entamoeba histolytica* HM1:IMSS is a virulent strain and *E. histolytica* Rahman is a non-virulent strain [7]. The HM1-IMSS strain was isolated in 1967 from a colonic biopsy of rectal ulcer from adult human male with amebic dysentery, Mexico City, Mexico. The HM1-IMSS was deposited in the American strain collection (ATCC® 30459™) and it is a gift of Professor Ruy Perez Tamayo (UNAM, Mexico). To maintain virulence, the HM1-IMSS strain has been passed through the liver of hamsters (Male Syrian golden hamsters *Mesocricetus auratus*) (roughly 174 passages since isolation until experiments were done). The procedure applied for animal infection was previously described [6], trophozoites were isolated from the liver abscesses after 7 days of intraportal inoculation (4 animals), mixed and further growth in axenic conditions. The Rahman strain is non-virulent [7] and is unable to growth in animals due to its inherent phenotype. The Rahman strain has been maintained in axenic culture since isolation in 1978 (with undetermined periods of frozen preservation) and it is a gift of Professor David Mirelman (Weizmann Institute, Israel). Trophozoites of both strains were grown axenically in TYI-S-33 medium at 37°C [37] and harvested during the exponential growth phase.

### Human colon explants preparation and histological analysis

Previous experimental published conditions were used for handling human colon pieces [5]. *Briefly*, 1.6×10^5^ trophozoites were added to the luminal face of the colon and incubated in Krebs buffer at 37°C for 1 and 7 h. After 1 h of incubation, mucus interacting trophozoites were collected by pipetting the mucus layer and 1 ml of Trizol was added. After 7 h of incubation, tissue fragments were fixed either in Carnoy fixative or in PFA (4%) and included into paraffin. PFA-fixed tissue sections were immunostained with a 1∶200 diluted rabbit antibody recognizing the Gal/GalNAc lectin [5] Sections from Carnoy-fixed tissue were stained with Alcian blue to visualize the mucus layer [38]. For each experiment, a representative histology image was taken.

### In-situ histological measurements of mucus thickness

For the measurement of mucus layer thickness, transverse sections were stained with Alcian blue stain. Light microscope images (NIKON, Eclipse E800) were analysed with ACT-1 software (NIKON). The mucus layer thickness was measured at three points of twenty different sections for three different patients (60 measurements for each condition). The mean of these measurements was considered as the mucus thickness for each condition. Statistical analysis was performed using GraphPad Prism software version 5.0b (GraphPad Software Inc). An unpaired, two-tailed student T-test was performed. Differences being considered as significant if P<0.05. Data are expressed as mean ± SEM.

### RNA isolation, cDNA synthesis, DNA chip hybridization and analysis

*Entamoeba histolytica* HM1:IMSS or Rahman trophozoites (1.6×10^5^) grown in axenic culture were lysed with Trizol reagent (Invitrogen), and total RNA isolated according to the manufacturer's protocol. RNA from mucus-interacting trophozoites was purified by gently scratching-off the mucus layer containing the trophozoites after 1 h of incubation. Trizol was added to the samples and RNA purification was performed. RNA was analysed for integrity and the concentration determined by capillary electrophoresis using the Agilent Bioanalyzer 2100 RNA nanochip Assay (Agilent Technologies). RNA from mucus-interacting trophozoites showed a mixture of amoebic and human RNA (up to 30%). Thus RNA isolated from human epithelial cells was used as a control to evaluate potential cross-hybridization of human transcripts in the subsequent experiments. Agilent microarrays EH-IP2008, scanning the entire amoebic genome, were used as previously described [17]. Six biological replicates were performed with amoebic strains grown in culture or incubated with the colon explants. Dye swap hybridizations were performed for the six biological replicates leading to a total of 12 hybridizations for each of the four conditions: Rahman in colon vs culture, HM1:IMSS in colon vs culture, Rahman vs HM1:IMSS in the colon, and Rahman vs HM1:IMSS in culture. In addition, one technical replicate was performed for one of the biological replicates and two self-self hybridizations were conducted. The resulting fluorescence signals were used to tune the scanner for the set of arrays. Probes cross-hybridizing to human RNA were identified and removed from the analysis (data not show). In addition, since prior to colon mucus contact the parasites were incubated in Krebs buffer we also determined gene expression changes in Krebs buffer; the modulated genes in each strain were removed before the analysis (Data in Table S1 and S2). The experiment finally yielded 54 competitive hybridizations. The whole data set was submitted to the ArrayExpress database (Accession number: E-MTAB-1201).

### Statistical analysis

A Principal Component Analysis of the whole microarray dataset was first carried out with Partek (http://www.partek.com/software) on the raw data. Microarray data statistical analyses were carried out with the R software (http://www.R-project.org) and Bioconductor packages (http://www.bioconductor.org). Our experiment follows a multifactorial design that includes two strains (HM1:IMSS and Rahman) in two different growth conditions (colon and culture). Linear models are well suited for the analysis of such designs, since they allow a global analysis of the whole dataset. Global effects, such as strain or growth condition effects can be measured, as well as differences between particular pairs of combinations of factors called contrasts, for example, the difference between Rahman and HM1:IMSS in colon condition. As Limma implements linear models for microarray data analysis, it was chosen for the present study (Limma package [19]). A Loess normalization was first performed on the 48 microarrays in order to render expression ratios comparable. The full experimental design was described through a design matrix (as explained in the Limma vignette) which is a binary matrix composed of (0, 1, −1) used by the linear model. The matrix makes a formal correspondence between arrays and pairs of conditions that have been hybridized. Then, a contrast matrix was created. It contains the list of comparisons that we wish to test with the linear model, namely HM1:IMSS – colon vs culture, Rahman – colon vs culture, HM1:IMSS vs Rahman – colon, HM1:IMSS vs Rahman – culture, HM1:IMSS vs Rahman, and colon vs culture. The moderated t-test associated with the empirical Bayes method (33was first applied to the hybridization value of each probe and the resulting p-values were further adjusted using a Bonferroni correction [39]). Finally, a median log-ratio was computed taking all probes in consideration in the case of genes represented by more than one probe on the array. An equivalent analysis was performed on a gene basis using the same design and contrast matrices and the same p-value adjustment. Only genes with an adjusted p-value lower than 0.05 and a fold change higher than 2 were considered for further analysis. Notice that according to this microarray analysis, upregulated and downregulated genes were taken into consideration. Thus the final fold changes values correspond to the ratio of changes between the two strains (i.e numbers from HM1:IMSS versus numbers from Rahman and vice versa). In other words genes appearing upregulated for HMI:IMSS strain are down regulated for Rahman strain counterpart and conversely genes upregulated for Rahman are downregulated for HM1:IMSS.

### Functional enrichment of genes upregulated in HM1:IMSS

Gene ontology and KEGG pathway annotations were retrieved from AmoebaDB v3.0 [40] and KEGG database [25]. To test for gene ontology enrichment, genes that are significantly upregulated (FDR<0.05, without fold-change cut-off) in HM1:IMSS comparing with Rahman during colon invasion were used as the foreground to test against the whole gene background using the hyper-geometric test implemented in FUNC package [23]. To test for KEGG pathway enrichment, the moderated fold-change of all genes in HM1:IMSS versus Rahman during colon invasion was used as the input into GSEA package [24]. Statistical significance was determined according to the default false discovery rates of the packages (5% in FUNC and 25% in GSEA).

### Bioinformatics analysis of β-amylase from *E. histolytica*

The structure of β-amylase from *E. histolytica* (Accession number EHI_192590) was predicted using LOMETS [41] which identifies β-amylase from *Glycine max* (Accession number: BMY1; 547931 BMY1) as the best-hit template (Z score = 102, 377). Protein domains were identified with SMART and defined EHI_192590 as a member of glycoside hydrolase family 14 which comprises β-amylase (EC 3.2.1.2). The amino acid sequence of the full-length homolog in *Entamoeba* was aligned by CLUSTALW software with β-amylase from *G. max*. The N-terminal tail of *E. histolytica* β-amylase was predicted as a transmembrane domain using TMHMM plugin of the Geneious software.

### dsRNA expression plasmids and RNA interference

To construct the dsRNA expression vectors, DNA fragments of the *E. histolytica* β-amylase gene (position +694 to position +1187, GenBank Accession number: EHI_192590) and the entire green fluorescent protein (GFP) coding sequence (GenBank Accession number: U73901) were amplified by PCR and subcloned into the TA-cloning vector pCR2.1-TOPO (Invitrogen). DNA inserts were excised from these constructs with restriction enzymes (KpnI and BamHI for GFP; KpnI and Bgl II for β-amylase) and cloned into the MCS of the L4440 plasmid vector that is bidirectionally flanked by T7 promoters. The resulting plasmids construct L4440-β-amylase and L4440-GFP were verified by restriction analysis and DNA sequencing. To purify dsRNA and perform soaking experiments we followed the procedure described previously [28].

### Antibodies and western blot analysis

A polyclonal rabbit anti-β-amylase antibody raised against the full-length β-amylase of *Ipomoea batatas* (sweet potato) was purchased from Abcam (ab6617). The specificity of this antibody was assessed by expression of amoebic β-amylase encoding gene (EHI_192590) in Escherichia coli (BL 21 strain). To this end the gene was amplified from the amoebic genome (forward primer: TACCATGGATGTTATTAACACTATGTTTTATATCAATAGC; reverse primer: ATCTCGAGTCTCATTGAATTAACAAATGAACAA) and cloned in MCS of pET28 vector. The insert was verified by DNA sequencing and upon expression in bacteria the recombinant protein was identified by western blot (Figure S2). For western blot analysis of amoebic extracts, the loaded protein amounts were normalized using an anti-actin C4 monoclonal antibody (ref: 08691001, MP Biomedicals) and secondary HRP-antibodies (MP Biomedicals) were used. Trophozoites submitted to dsRNA soaking experiments were collected to prepare crude extracts as previously described [42]. Crude extracts (4×10^4^ cells/lane) were resolved by SDS-PAGE, transferred to PVDF membranes and incubated with specific antibodies and ECL Plus reagent (GE Healthcare Bio-sciences) for chemiluminescence detection. Semi-quantitative analysis of light emission from probed nitrocellulose membranes was carried out from scanned autoradiographs using Quantity one software (BioRad) and protein abundance was normalized with actin values.

### Immunofluorescence and confocal microscopy analysis

Trophozoites were grown axenically in TYI-S-33 medium at 37°C and then centrifuged for 5 min at 550× *g* during the exponential growth phase. The pellet was fixed in 4% paraformaldehyde at 37°C for 15 min and permeabilized or not with Triton X-100. Cells were incubated in 1% PBS/BSA to avoid non-specific labelling. The primary antibody againstβ-amylase 1/1000 (Abcam® (ab6617)) was then deposited onto the coverslip and incubated in a humid chamber for 2 h at 37°C. The coverslips were washed in 1% PBS/BSA and the secondary antibody coupled to Alexa-568 (Molecular Probes, Invitrogen) 1/200 was added to the coverslip and incubated in a humid chamber for 30 min at 37°C. The coverslips were washed and the slides were then mounted using VectaShield mounting medium, sealed and conserved at 4°C until confocal microscopy analysis. The slides were analysed using a Zeiss LSM 710 Confocal Microscope and LSM software.

## Supporting Information

Figure S1 Western blot analysis of the recombinant β-amylase expressed in *E. Coli*. The upper panel show the recombinant β-amylase revealed by the anti β-amylase raised against the full-length β-amylase of *Ipomoea batatas* (sweet potato). The lower panel show the recombinant β-amylase revealed by the anti 6×-his tag. Lane 1: *E. histolytica* crude extract; Lane 2: *E. coli*, BL 21 strain crude extract; Lane 3: *E. coli*, BL21 strain expressing the amoebic β-amylase (+IPTG); Lane 4: *E. coli*, BL21 strain non-induced (−IPTG).
(TIF)

Figure S2 A. Amino acid sequence alignment of the 8 full-length β-amylase homologues in *Entamoeba histolytica* (EHI_009020, EHI_035700, EHI_049700, EHI_148800, EHI_053840, EHI_118440, EHI_192590 and EHI_098200) revealed 76.7% of pairwise identity and 40.7% of identical sites. The red line indicates the sequence used to design the dsRNA. B. Column B and C respectively show RNASeq data expressed in fragment per kilobase per millon reads (FPKM) of the 8 β-amylase alleles in Rahman or in HM1:IMSS under axenic culture. Note that in HM1:IMSS, EHI_192590 account for more than 80% of the β-amylase transcripts and that all these genes are almost non-expressed in Rahman in axenic culture.
(TIF)

Table S1 Genes showing modulated expression in HM1:IMSS after contact with Krebs media compared to axenic culture growth. FC: Fold Change; BF: Bonferroni adjusted p value≤0.05.
(XLSX)

Table S2 Genes showing modulated expression in Rahman after contact with Krebs media compared to axenic culture growth. FC: Fold Change; BF: Bonferroni adjusted p value≤0.05.
(XLS)

Table S3 Genes showing modulated expression in HM1:IMSS compared to Rahman under axenic culture growth. FC: Fold Change; BF: Bonferroni adjusted p value≤0.05.
(XLS)

Table S4 Genes showing modulated expression in HM1:IMSS after contact with human colon explant compared to axenic culture growth. FC: Fold Change; BF: Bonferroni adjusted p value≤0.05.
(XLS)

Table S5 Genes showing modulated expression in Rahman after contact with human colon explant compared to axenic culture growth. FC: Fold Change; BF: Bonferroni adjusted p value≤0.05.
(XLS)

Table S6 Genes showing modulated expression in HM1:IMSS compared to Rahman after contact with human colon explant. FC: Fold Change; BF: Bonferroni adjusted p value≤0.05.
(XLS)

Table S7 GO term enrichment analysis. Carbohydrate metabolism related gene ontology terms are highlighted in RED.
(XLS)

Table S8 GSEA analysis of the KEGG pathway. Carbohydrate metabolism related gene ontology terms are highlighted in RED.
(XLS)

Table S9 Genes significantly up-regulated in HM1:IMSS with a fold change lower than two. FC: Fold Change; BF: Bonferroni adjusted p value≤0.05.
(XLS)

Video S1 Two photon video-microscopy of Rahman trophozoites migrating at the surface of the mucus layer after 7 h of incubation with human colon explant. Trophozoites were stained with a red cell tracker and visualised using an excitation output wavelength at 820 nm. The detection bandwidth was 570–610 nm. Signals were collected with a backscattering geometry and non-descanned. The xy plane images (512 * 512 pixels per frame, acquired in 6.71 seconds).
(AVI)
