# Supplementary figures and images for: Identification of the Virulence Landscape Essential for Entamoeba histolytica Invasion of the Human Colon
Source: PLoS Pathog. 2013 Dec 19;9(12):e1003824. doi: 10.1371/journal.ppat.1003824 (PMC3868522; doi:10.1371/journal.ppat.1003824)

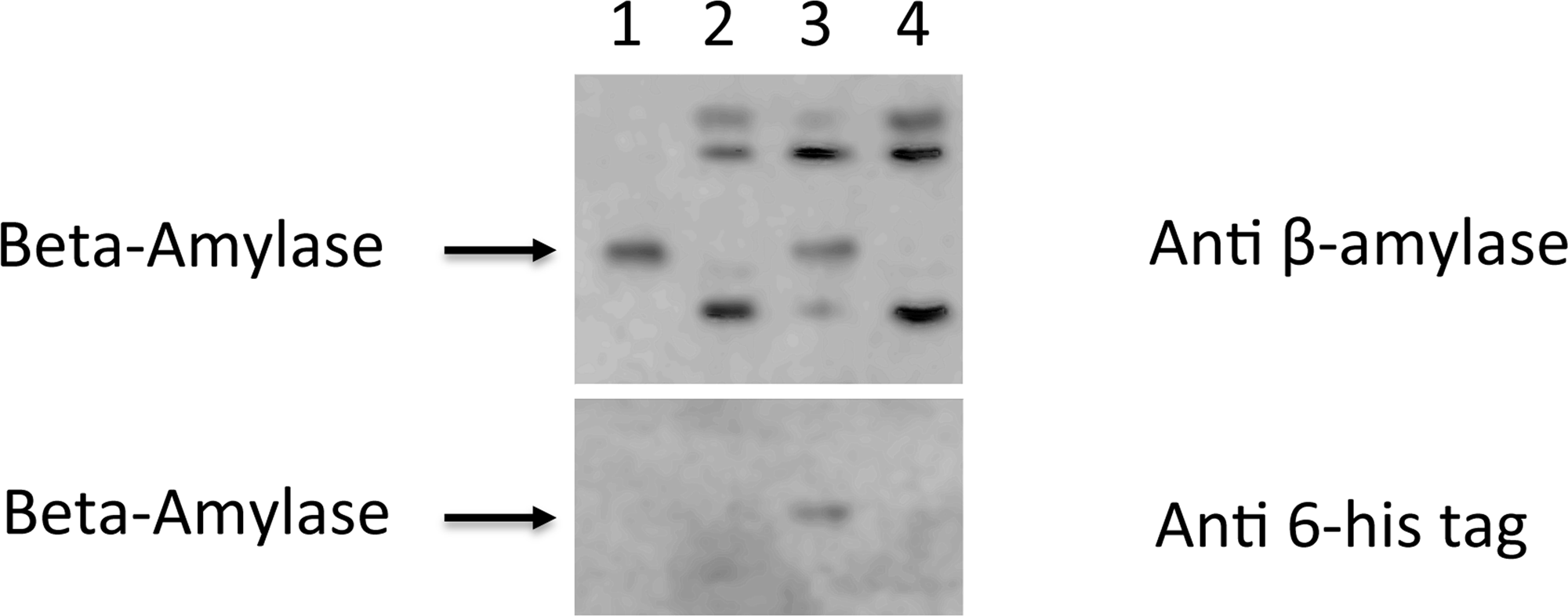

Supplement: Figure S1 — Western blot analysis of the recombinant β-amylase expressed in E. Coli. The upper panel show the recombinant β-amylase revealed by the anti β-amylase raised against the full-length β-amylase of Ipomoea batatas (sweet potato). The lower panel show the recombinant β-amylase revealed by the anti 6×-his tag. Lane 1: E. histolytica crude extract; Lane 2: E. coli, BL 21 strain crude extract; Lane 3: E. coli, BL21 strain expressing the amoebic β-amylase (+IPTG); Lane 4: E. coli, BL21 strain non-induced (−IPTG). (TIF) [file ppat.1003824.s001.tif]

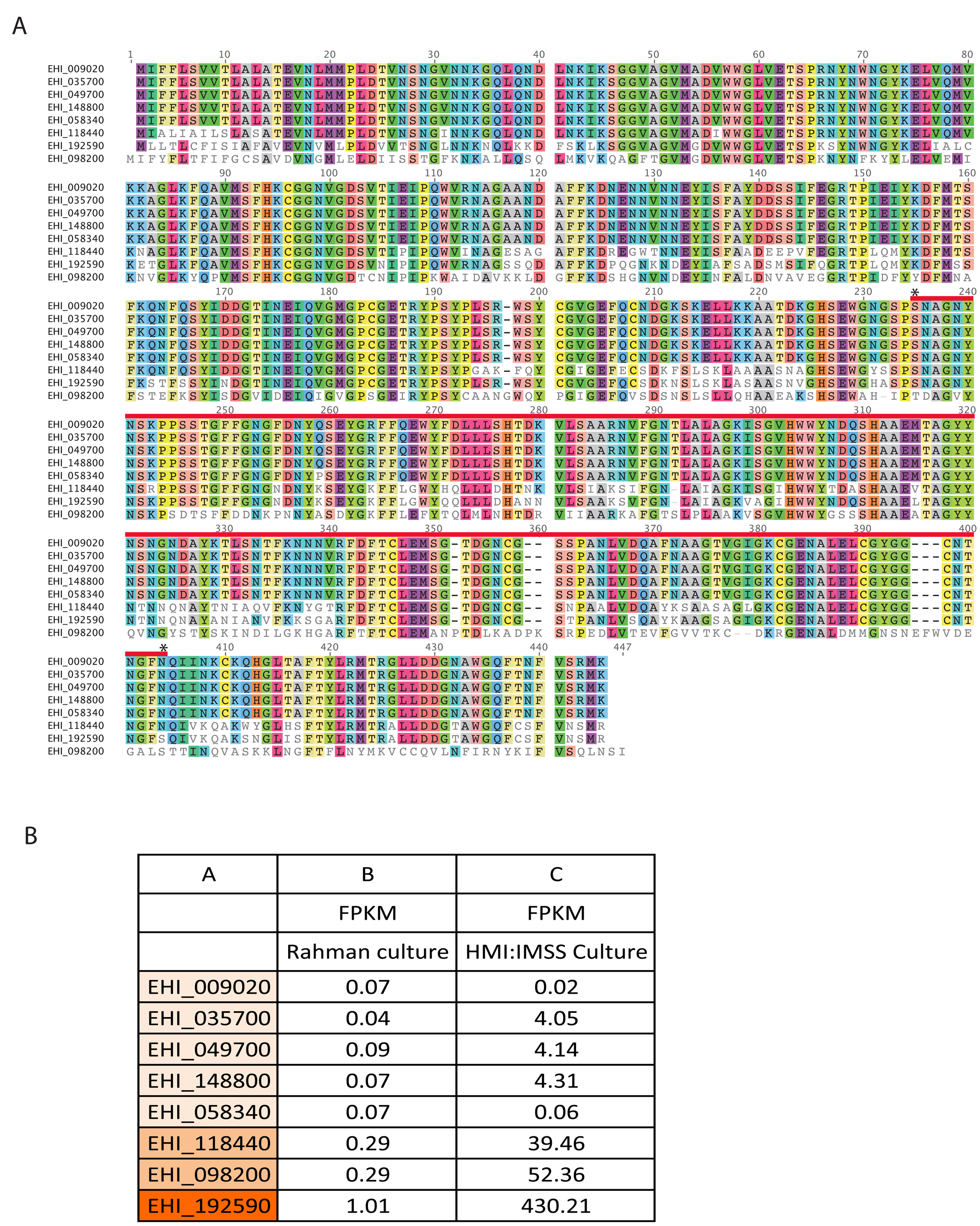

Supplement: Figure S2 — A. Amino acid sequence alignment of the 8 full-length β-amylase homologues in Entamoeba histolytica (EHI_009020, EHI_035700, EHI_049700, EHI_148800, EHI_053840, EHI_118440, EHI_192590 and EHI_098200) revealed 76.7% of pairwise identity and 40.7% of identical sites. The red line indicates the sequence used to design the dsRNA. B. Column B and C respectively show RNASeq data expressed in fragment per kilobase per millon reads (FPKM) of the 8 β-amylase alleles in Rahman or in HM1:IMSS under axenic culture. Note that in HM1:IMSS, EHI_192590 account for more than 80% of the β-amylase transcripts and that all these genes are almost non-expressed in Rahman in axenic culture. (TIF) [file ppat.1003824.s002.tif]
